# Rejuvenation of mesenchymal stem cells by human peripheral blood lymphocytes

**DOI:** 10.1186/s12915-025-02472-9

**Published:** 2025-11-25

**Authors:** Yi Luo, Xin-Xin Zhu, Qing-Rong Le, Wen-Ting Chen, Yan Xu, Xue-Mei Chen, Huan Yuan, Xu Yang, Jun-Wei Xu, Jian-Jiang Zhong, Jian-Hui Xiao

**Affiliations:** 1 Institute of Medicinal Biotechnology & Center for Translational Medicine, Affiliated Hospital of Zunyi Medical University, 149 Dalian Road, Huichuan District, Zunyi, 563003 China; 2 Faculty of Life Science and Technology, Kunming University of Science and Technology, Kunming, 650500 China; 3 State Key Laboratory of Microbial Metabolism, and School of Life Sciences & Biotechnology, Shanghai Jiao Tong University, 800 Dongchuan Road, Shanghai, 200240 China; 4 Guizhou Provincial Key Laboratory of Medicinal Biotechnology & Research Center for Translational Medicine in Colleges and Universities, Affiliated Hospital of Zunyi Medical University, 149 Dalian Road, Huichuan District, Zunyi, 563003 China; 5 Department of Pediatrics, Affiliated Hospital of Zunyi Medical University, 149 Dalian Road, Huichuan District, Zunyi, 563003 China

**Keywords:** Mesenchymal stem cell, Peripheral blood lymphocytes, In vitro coculture expansion system, Anti-senescence, Cell proliferation

## Abstract

**Background:**

In the in vitro expansion of mesenchymal stem cells (MSCs), replicative or stress-induced senescence poses a significant challenge, leading to the loss of their cellular properties and therapeutic functions. Currently, there is a lack of efficient strategies to address this issue.

**Results:**

Here we presented a novel approach to combat cellular senescence and promote cell proliferation by coculturing human MSCs with human peripheral blood lymphocytes (PBLs). In a heterogeneous population of MSCs comprising both aged and nonaged cells, PBL effector cells, rather than their cytokines, exhibited a dual role. They selectively induced apoptosis in aged cells by facilitating p53 SUMOylation and activating the p53-dependent mitochondrial pathway, while simultaneously safeguarding younger cells against senescence and promoting cell proliferation by activating Serpinb2/NF-κB signaling. This resulted in a decrease in aged MSCs and an enrichment of rejuvenated MSCs. This process effectively reversed the senescence phenotype, leading to the remodeling of stemness characteristics and the enhancement of functionality within the MSC population. Furthermore, MSCs rejuvenated by PBLs presented an enhanced therapeutic efficacy and a favorable safety profile.

**Conclusions:**

PBLs rejuvenate MSCs by promptly removing aged cells and enhancing the stemness and proliferative capacity of nonaged cells. This work provides an ideal method for obtaining substantial MSCs while meeting the quality requirements for stem cell therapy.

**Graphical Abstract:**

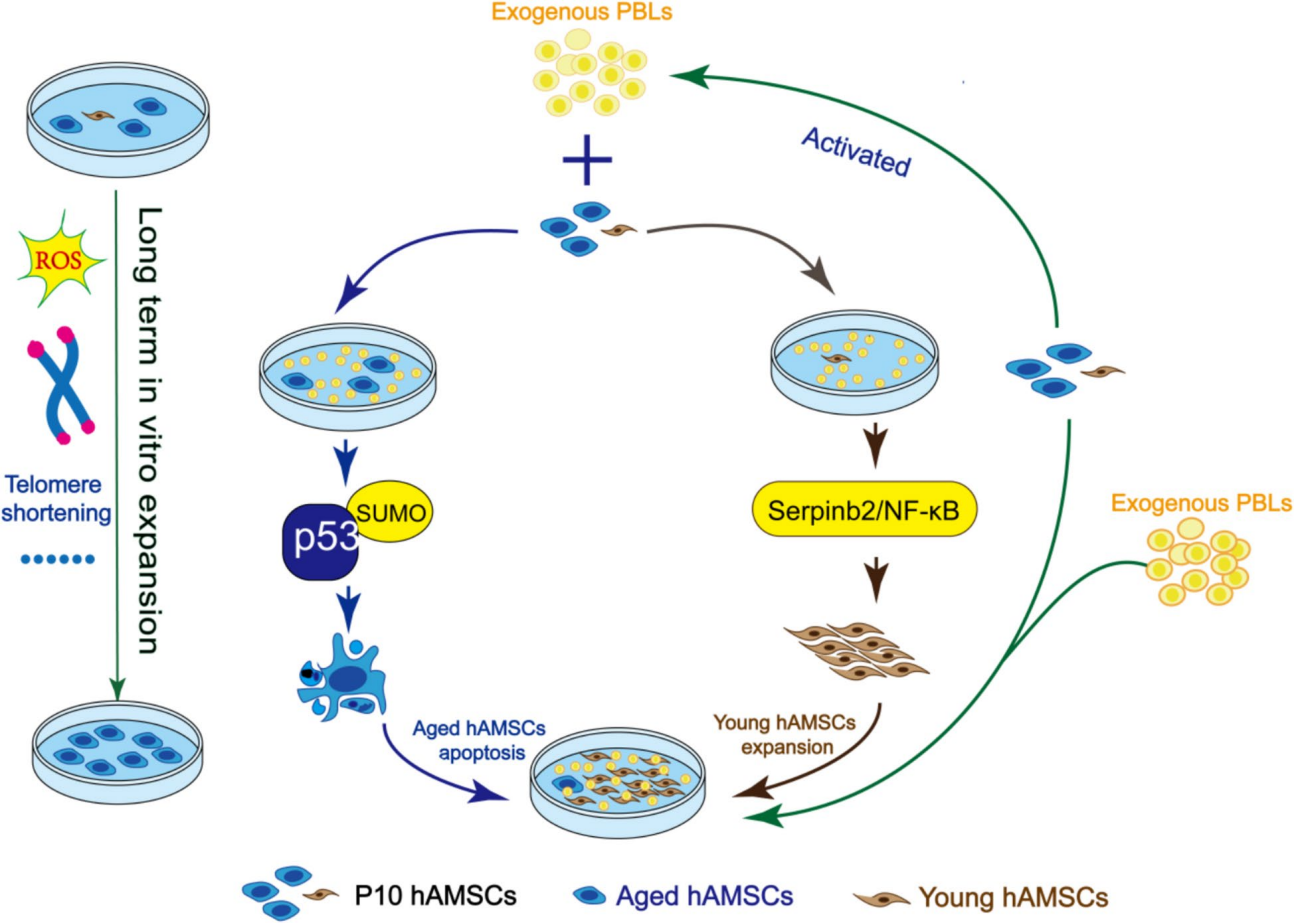

**Supplementary Information:**

The online version contains supplementary material available at 10.1186/s12915-025-02472-9.

## Background

Stem cell therapy represents a groundbreaking approach for addressing numerous incurable diseases [1, 2]. Mesenchymal stem cells (MSCs) stand out as highly promising sources for regenerative medicine, owing to their regenerative properties and ability to restore tissue homeostasis postinjury [3]. The number of MSC-based clinical trials has surged in recent years, with over 1500 clinical trials registered as completed or ongoing according to the ClinicalTrials.gov database. Clinical studies have demonstrated that the effective dose range for MSC infusion is 1–2 × 10^6^ cells per kg body weight, which are administered 1–10 times per trial [4]. Thus, obtaining a sufficient number of MSCs is crucial, necessitating in vitro expansion of MSCs isolated from specific tissues prior to clinical use [5].

However, during in vitro cultivation, prolonged cell division, genomic damage, and various stress stimuli exacerbate MSC heterogeneity and severely impair cell quality, significantly weakening MSC functionality and availability [6, 7]. For example, in vitro expansion leads to dramatic changes in the transcriptome and proteome expression profiles, reducing the proliferation and differentiation potential of MSCs [8]. Furthermore, environmental factors and oxidative stress contribute to the Hayflick limit and senescence during in vitro expansion [9], resulting in the progressive loss of key biological characteristics in MSCs, such as self-renewal, differentiation potential, immune regulation, and migration [10–12]. This cellular disability is aggravated by the senescence-associated secretory phenotype (SASP) produced by senescent cells, which can induce neighboring normal cells to enter a senescent state [13, 14]. Notably, aged MSC infusion may elevate the risk of immune rejection and tumor formation [11, 12]. Therefore, long-term in vitro expansion triggering cellular senescence has emerged as a significant limitation in the clinical application of MSCs.

Addressing MSC senescence during prolonged in vitro expansion and achieving a substantial quantity of functional MSCs has become a focal point in regenerative medicine. Various strategies, including genetic reprogramming [15, 16], cytokine stimulation [17], chemical regulation [18], and biomaterial interaction [19], have been reported to combat senescence and improve cellular quality. However, these approaches have drawbacks, such as tumorigenesis, off-target effects, immune rejection, high cost, and limited effectiveness. Well-known senotherapeutics that target senescent cells, including quercetin, dasatinib, and fisetin alone or in combination, have shown efficacy in promoting senescent cell death in vivo [20, 21]. Our recent research demonstrated that ganoderic acid D effectively delayed the senescence of human amniotic membrane MSCs (hAMSCs) in vitro [22, 23]. However, these methods have limitations in promoting cell division to obtain a sufficient number of functional stem cells. Recent advancements, particularly in mimicking the in vivo stem cell microenvironment, have shown promise in bolstering cellular function [9, 24, 25]. Targeting MSC heterogeneity within the expansion system may effectively combat MSC senescence by promptly removing aged cells and enhancing the stemness of nonaged cells. However, such an approach has not yet been attempted.

Senescent cells within healthy organisms are highly immunogenic and subject to immune surveillance and immune clearance by the host immune system [26]. Interestingly, activated and expanded natural killer (NK) cells derived from both patients and healthy individuals and subsequently autologously transfused intravenously can notably decrease the levels of senescence markers such as p16 and senescence-associated β-galactosidase (SA-β-gal) in peripheral blood mononuclear cells. This process improves the senescence and depletion state of T cells and reduces the levels of SASP factors such as IL-6, IL-8, and IFN-γ [27, 28]. These findings suggest that the activation of autologous immune cells may alleviate immune senescence and mitigate senescence-associated inflammatory responses. The role of activated autoimmune cells in preventing senescence in organisms is unquestionable. However, it remains unknown whether nonactivated allogeneic human immune cells can recognize and clear aged MSCs within an in vitro amplification system.

In this study, we utilized human peripheral blood-derived lymphocytes (PBLs) and hAMSCs as experimental subjects to explore the effects of PBLs on MSC senescence within an in vitro amplification system. We investigated whether hAMSC heterogeneity and senescence within the expansion system could be combated by this novel PBL-hAMSC coculture approach. Furthermore, we assessed the safety and quality of functional MSCs obtained through coculture with human PBLs in in vivo experiments.

## Results

### PBLs protected MSCs against senescence

**Fig. 1:**
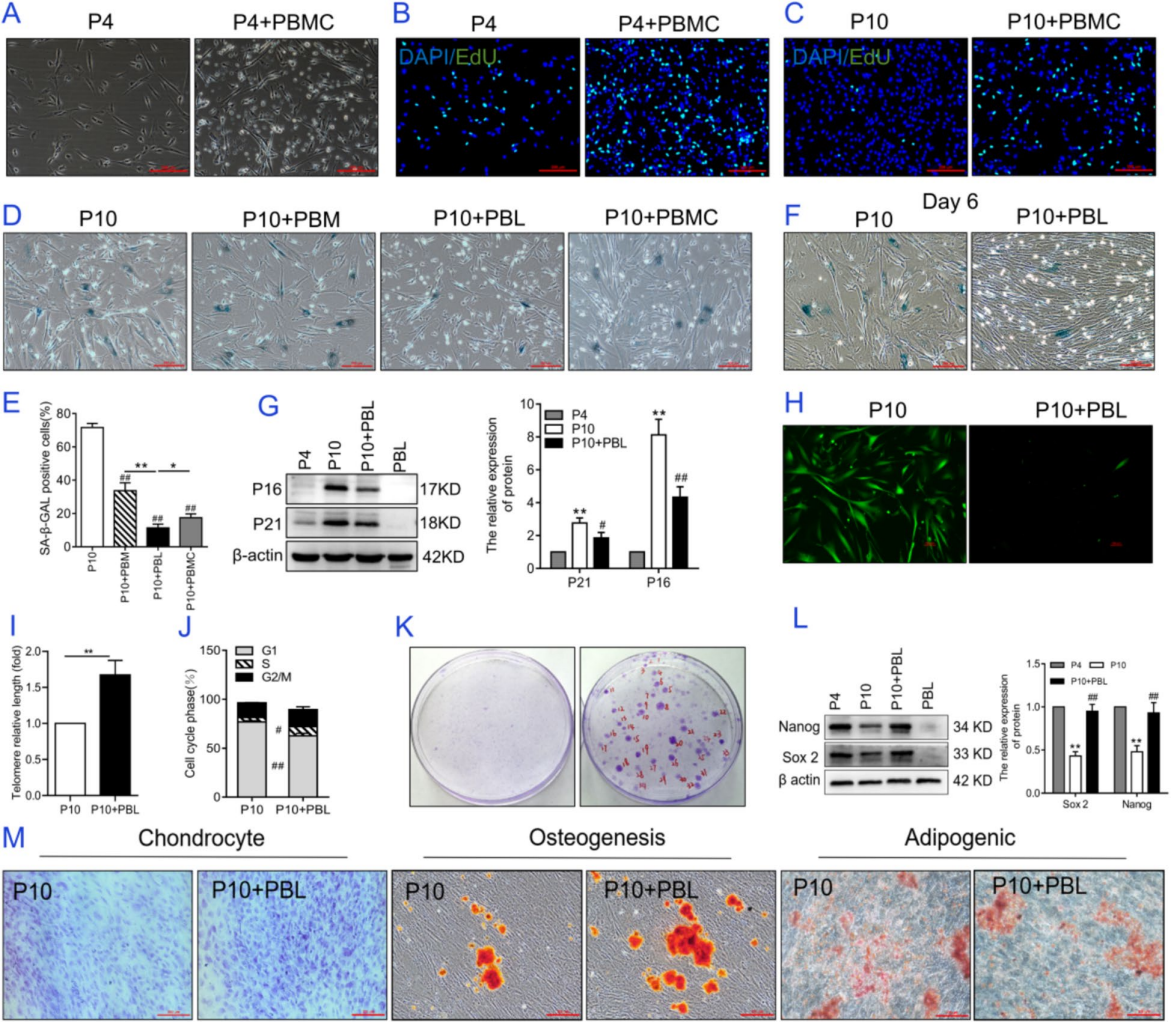
PBLs protect MSCs against senescence. **A** Morphological analysis of young hAMSCs after coculture with PBMCs for 48 h. **B**, **C** EdU analysis of proliferation in **B** P4 hAMSCs and **C** P10 hAMSCs. **D** Analysis of SA-β-gal staining in P10 hAMSCs. P10 hAMSCs were cultured with PBMs, PBLs, or PBMCs for 3 days. **E** Quantitative statistical analysis of the data in **D**. **F** Analysis of SA-β-gal staining in P10 hAMSCs. P10 hAMSCs were cultured with PBLs for 6 days. **G** Analysis of P21 and P16 protein expression levels in P10 hAMSCs. **H** ROS levels in P10 hAMSCs. Scale bar: 100 µm. **I** Relative telomere length in P10 hAMSCs was analyzed via qPCR. **J** Cell cycle analysis of P10 hAMSCs. (K) Cell colony formation ability of P10 hAMSCs. **L** Analysis of Sox2 and Nanog protein expression levels in P10 hAMSCs. **M** Analysis of the ability of P10 hAMSCs to differentiate into chondrocytes, osteoblasts, and adipocytes. Scale bar: 100 µm. Note: P4: hAMSCs were expanded in vitro up to the fourth generation; P4 + PBL: hAMSCs from the fourth passage were cocultured with PBLs at a ratio of 1:100. P10: hAMSCs were expanded in vitro up to the tenth generation; P10 + PBL: hAMSCs from the tenth passage were cocultured with PBL at a ratio of 1:100. PBMCs: human peripheral blood-derived mononuclear cells; PBMs, human peripheral blood-derived monocytes; PBLs, human peripheral blood-derived lymphocytes; SA-β-gal, β-galactosidase. The data are presented as means ± SDs (*n* = 3). Statistical significance compared with the P4 group is denoted as ^**^P < 0.01; that compared with the P10 group is denoted as ^#^*P* < 0.05, ^##^*P* < 0.01

The dynamic interplay between the stem cells and immune microenvironments has been observed in disease pathogenesis, particularly in tumor immunobiology [29]. While in vitro studies demonstrated immune cell-mediated modulation of MSC behavior, current findings reveal significant context-dependent variations. Unstimulated PBMCs generally exhibited limited impact on MSC proliferation, though supraphysiological PBMC-to-MSC ratios (e.g., 25:1) demonstrated marked growth suppression [30, 31]. Paradoxically, adipose-derived MSCs displayed mild proliferative enhancement during PBMC coculture [32]. In this study, fresh human peripheral blood mononuclear cells (PBMCs) were isolated as providers of the immune microenvironment and then were cocultured with young hAMSCs (P4 hAMSCs) in vitro. This coculture system strongly stimulated the proliferation of hAMSCs and increased the number of EDU-positive cells (Fig. 1A, B). Additionally, PBMCs from different individual sources displayed consistent pro-proliferative characteristics (Additional file 1: Fig. S1A). Next, 10th generation hAMSCs (P10 hAMSCs), containing approximately 50 to 70% aged cells, were cocultured with PBMCs, and the proliferation ability of P10 hAMSCs was also enhanced (Fig. 1C). More intriguingly, the number of SA-β-gal-positive cells, a recognized cellular marker of senescence, was reduced, and this event was independent of the source of the PBMC donors (Additional file 1: Fig. S1B). These data suggest that the immune microenvironment provided by PBMCs can reverse the senescence phenotype of hAMSCs while promoting hAMSC division in the coculture system. PBMCs are a complex cell population that briefly includes peripheral blood lymphocytes (PBLs) and peripheral blood monocytes (PBMs). Coculture with different types of human peripheral blood immune cells, including PBMs, PBLs, and PBMCs, could reduce the number of SA-β-gal-positive cells in P10 hAMSCs. In terms of the inhibitory rate of SA-β-gal-positive cells, PBL > PBMC > PBM, and PBL had the best anti-senescence effect (Fig. 1D, E). It is widely accepted that aged MSCs exhibit hypertrophic and flat cell morphology, while young MSCs display a fibroblast-like spindle shape and exhibit swirling growth patterns. Over a 72-h period, we monitored the cell growth and morphological changes in P10 hAMSCs within this coculture system in real time (Additional file 2: Videos S1-S2). Besides, PBLs reduced the number of SA-β-gal-positive cells in P10 hAMSCs in a time-dependent manner (Fig. 1F). Furthermore, PBL treatment also significantly ameliorated the senescent phenotype of MSCs derived from umbilical cord and umbilical cord blood (*P* < 0.01) (Additional file 1: Fig. S1C-D). It is widely accepted that aged MSCs exhibit hypertrophic and flat cell morphology, whereas young MSCs display a fibroblast-like spindle shape and exhibit swirling growth patterns. Based on this, Fig. 1F shows that the coculture system reduced the number of aged P10 hAMSCs and increased the number of young cells. In addition, the protein levels of the senescence markers p21 and p16, which are typically elevated in senescent cells, were significantly lower following PBL treatment than they were in P4 hAMSCs (*P* < 0.01), with PBLs significantly reducing the protein expression levels of p21 and p16 (*P* < 0.05 and *P* < 0.01, respectively; Fig. 1G). Furthermore, PBLs sharply reduced the formation of reactive oxygen species (ROS) (Fig. 1H) and increased the relative length of telomeres (Fig. 1I) in P10 hAMSCs. Senescent cells generally undergo irreversible cell cycle arrest in the G1 phase, and these data demonstrated that PBLs reversed the growth arrest of P10 hAMSCs by increasing the proportion of cells in the S phase and decreasing the proportion of cells in the G1 phase (Fig. 1J).

Self-renewal and multidirectional differentiation potential are negatively correlated with the degree of senescence of MSCs [33]. Notably, the coculture system enhanced the self-renewal ability and stemness of P10 hAMSCs. Key indicators of self-renewal ability, such as the colony formation rate, were significantly elevated following PBL treatment (Fig. 1K). Similarly, the expression levels of the stemness transcription factors Nanog and Sox2 in P10 hAMSCs were significantly increased in the coculture system, approaching to those of young P4 hAMSCs (*P* < 0.01; Fig. 1L). Furthermore, PBL treatment selectively promoted osteogenic and chondrogenic differentiation of hAMSCs while exerted negligible effects on adipogenic commitment, as shown in Fig. 1M.

### Activation and altered secretion profile of PBLs cocultured with MSCs

**Fig. 2:**
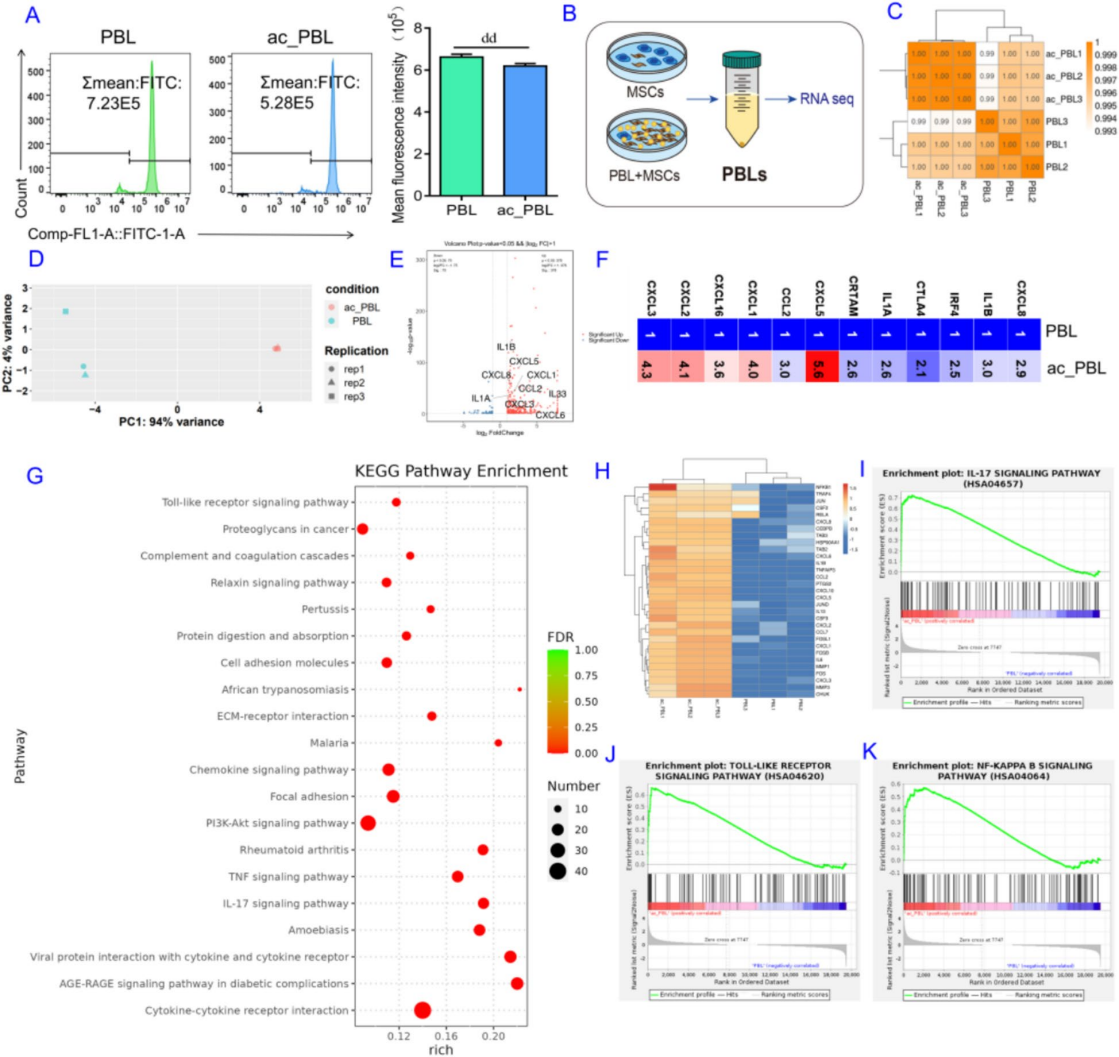
Coculture with MSCs activated PBLs, altering the secretion profile of PBLs. **A** Proliferation of PBLs was measured by flow cytometry after labeling with CFSE. **B** PBLs were harvested with or without coculture with hAMSCs for RNA-seq analysis. **C** Sample correlation test in RNA-seq. **D** Principal component analysis of the RNA-seq data. **E** Volcano plot of differentially expressed genes identified via RNA-seq. **F** Differential multiples of cytokine expression. **G** Clustering of differentially expressed genes in the IL-17 signaling pathway. **H** Analysis of the top 20 enriched KEGG items for the DEGs. **I**–**K** GSEA enrichment analysis of the RNA-seq data: **I** IL-17 signaling pathway; **J** Toll-like receptor signaling pathway; **K** NF-kappa B signaling pathway. Note: CFSE, carboxyfluorescein succinimidyl ester; PBL, lymphocytes freshly isolated from human peripheral blood; ac_PBL, PBLs after 12 h of coculture with P10 hAMSCs. The data are presented as the means ± SDs (*n* = 3). Statistical significance compared with the PBL group is denoted as.^dd^*P* < 0.01

Typically, PBLs require stimulation by antigenic substances to activate, proliferate, differentiate, and generate many specialized immune cells or immune effector cells [34], which can secrete various effector molecules or cytokines to regulate the behavior of MSCs. As shown in Fig. 2A, the coculture system significantly enhanced PBLs’ proliferation compared to the PBLs alone control at 12 h, indicating activation-triggered cytokine secretion that subsequently modulated MSCs’ behavior. To explore these factors regulating the behavior of P10 hAMSCs in PBLs, PBLs cocultured with or without hAMSCs were harvested for RNA sequencing (RNA-seq) (Fig. 2B). Three independent replicate samples from the PBL and activated PBL (ac_PBL) groups were sequenced, which revealed a high degree of consistency within each group (Fig. 2C, D). Pairwise comparisons between the PBL and ac_PBL groups identified differentially expressed genes (DEGs) induced by hAMSC treatment. Using custom normalization and differential analysis, 453 DEGs were identified with a |log2-fold change |> 1 and a significance *P*-value < 0.05 as the criteria. Specifically, 378 upregulated DEGs and 75 downregulated DEGs were detected in the ac_PBL group compared with the PBL group (Fig. 2E). The upregulated DEGs included inflammatory cytokines such as CXCL1, CXCL5, CXCL8, and IL-1β (Fig. 2E). The magnitude of the changes in inflammatory factors is shown in Fig. 2F. These data suggest that this coculture system triggers a strong cellular response in PBLs. KEGG enrichment analysis of the DEGs revealed that the top 20 pathways included the Toll-like receptor signaling pathway, the TNF signaling pathway, and the IL-17 signaling pathway (Fig. 2H). Figure G shows the DEGs involved in the IL-17 signaling pathway. We used gene set enrichment analysis (GSEA) to comprehensively reveal the effects of the DEGs produced by this coculture system on the cellular pathways involved in PBLs. Among the top 20 enriched KEGG pathways, the IL-17 signaling pathway, Toll-like receptor signaling pathway, and NF-kappa B signaling pathway were upregulated in the ac_PBL group (Fig. 2I–K). Heatmaps indicated that CXCL-type chemokines and IL-type cytokines in these pathways were significantly upregulated in the ac_PBL group (Additional file 1: Fig. S2). These data suggest that inflammatory signaling is the dominant cellular pathway in PBLs induced by hAMSCs. However, the effects of these upregulated cytokines on hAMSC phenotypes need to be further investigated.

### PBL effector cells rather than cytokines play a major role in reversing the senescent phenotype of MSCs

As mentioned above, P10 hAMSCs significantly altered the secretion profile of PBLs in this coculture system. Immune effector cells and effector molecules (cytokines) are mainly responsible for the onset of immune reactions [35]. To understand which immune effector substances, PBL effector cells or cytokines, play a major role in reversing hAMSC senescence, we analyzed the effects of the above upregulated cytokines, including CXCL1, CXCL8, CCL2, IL-33, IL-1α, and IL-1β, in the ac_PBL group on the morphology and SA-β-gal staining of P10 hAMSCs. These cytokine treatments did not mitigate the senescence phenotype of P10 hAMSCs (Additional file 1: Fig. S3A-B). In contrast, PBL treatment had superior anti-senescence effects on P10 hAMSCs (Additional file 1: Fig. S3A-B). However, compared with the P10 group, both the IL-1α and IL-1β groups presented increased cell counts and promoted P10 hAMSC division (Additional file 1: Fig. S3A). Although similar effects on cell proliferation were also observed in young hAMSCs (P4 hAMSCs), they were not as effective as PBL treatment (Additional file 1: Fig. S3C-E). As shown in Additional file 1: Fig. S3F, transwell assays revealed that direct contact coculture exerted superior rejuvenation effects on aged MSCs versus transwell-separated conditions (*P* < 0.01), suggesting physical cell–cell interactions potentiate the anti-senescence mechanisms. Thus, we concluded that PBL treatment could play a dual role in promoting proliferation and counteracting senescence in hAMSCs and that PBL effector cells, rather than cytokines, play a major role in reversing hAMSC senescence.

**Fig. 3:**
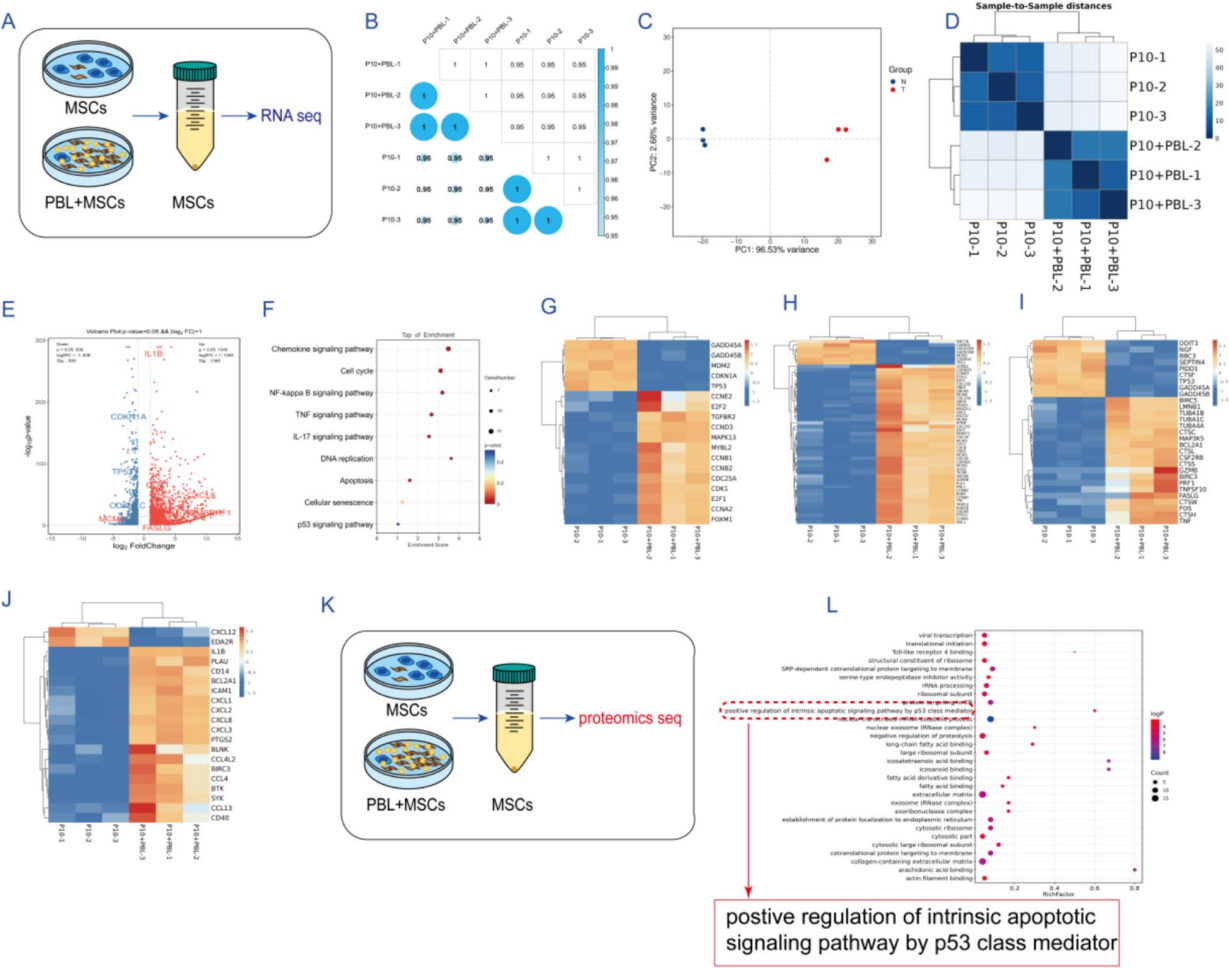
Effect of PBLs on the transcriptome and proteome of hAMSCs. **A** hAMSCs were harvested with or without coculture with PBLs for RNA-seq analysis. **B** Heatmap of the correlation coefficient between samples. **C** Principal component analysis of the RNA-seq data. **D** Sample-to-sample cluster analysis. **E** Volcano plot of the differentially expressed genes. **F** KEGG enrichment analysis of differentially expressed genes. **G**–**J** Clustering heatmaps of differentially expressed genes related to **G** cellular senescence, **H** the cell cycle, **I** apoptosis, and **J** the NF-κB signaling pathway. **K** hAMSCs were harvested with or without coculture with PBLs for proteomics-seq analysis. **L** GO enrichment analysis of differentially expressed proteins

To understand the underlying mechanism by which PBLs reverse hAMSC senescence, we used RNA-seq analysis to determine the transcriptomic changes induced by PBL treatment in P10 hAMSCs (Fig. 3A), to provide a basis for subsequent studies. Three independent replicates of the P10 and P10 + PBL groups were sequenced, and the replicates of each group presented a high degree of concordance (Fig. 3B). Pairwise comparisons between the P10 and P10_PBL groups revealed DEGs in hAMSCs following PBL treatment. According to the criteria for DEGs, 2184 genes were identified, 1346 of which were upregulated and 838 of which were downregulated in the P10_PBL group compared with the P10 group (Fig. 3C–E). These data suggest that PBL treatment triggers a robust cellular response in hAMSCs. KEGG enrichment analysis revealed that the top 20 enriched pathways included the cell cycle, p53 signaling pathway, phagosome in cellular processes, the chemokine signaling pathway, longevity-regulating pathway, and PI3K-Akt signaling pathway (Additional file 1: Fig. S4A-C). Given our previous findings, the effects of this coculture system affect hAMSC behavior, including anti-senescence, enhancing proliferative capacity, and cytokine secretion. Therefore, in the KEGG enrichment terms, we focused on the enrichment of the cell cycle, apoptosis, and inflammatory signaling pathways (Fig. 3F). Figure 3G–J show the changes in the transcription levels of genes related to the cell cycle, cellular senescence, apoptosis, and NF-κB signaling pathways. Next, we used iTRAQ quantitative proteomics-seq analysis to determine the proteomic changes induced by PBL treatment in P10 hAMSCs (Fig. 3K). In GO analysis, it was revealed that positive regulation of the intrinsic apoptotic signaling pathway by p53 class mediators was enriched. This finding is consistent with the enrichment of apoptotic and p53 signals in the RNA-seq data (Additional file 1: Fig. S3A, Fig. 3H). On the basis of these findings, we hypothesized that this coculture system may induce the apoptosis of aged cells in P10 hAMSCs through positively regulating the P53-dependent endogenous mitochondrial apoptotic pathway, thereby reducing the number of aged cells.

### Activated PBLs targeted and induced the apoptosis of aged MSCs through the p53-dependent mitochondrial pathway

**Fig. 4:**
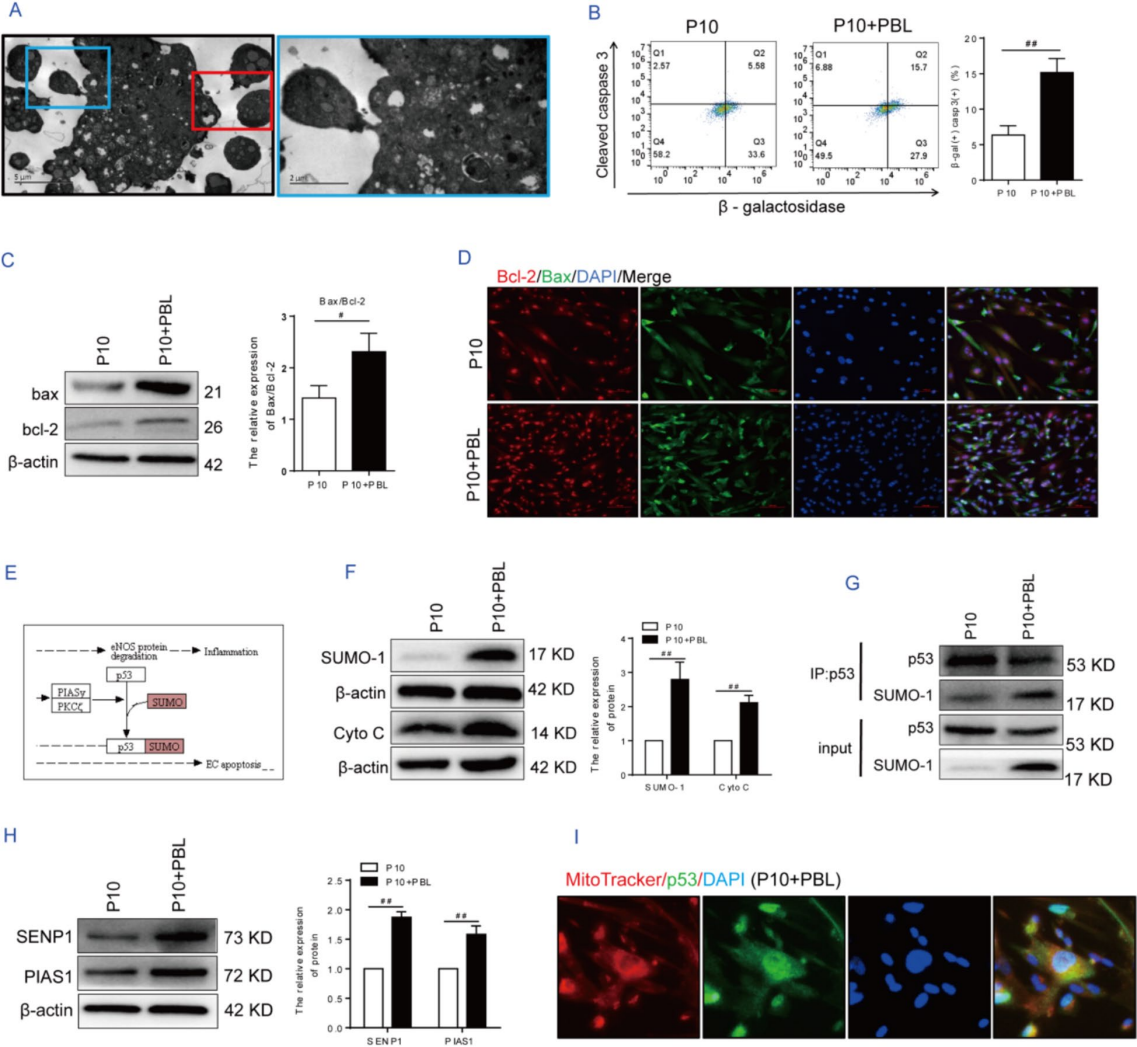
Activated PBLs targeted and induced the apoptosis of aged MSCs through the p53-dependent mitochondrial pathway. **A** The apoptotic bodies in the P10 + PBL group were analyzed by scanning electron microscopy. **B** SA-β-gal and cleaved caspase 3 double-staining analysis of PBL-targeted induction of apoptosis in senescent cells. **C** The Bax/Bcl-2 ratio at the protein level. **D** Immunofluorescence analysis of Bax and Bcl-2 protein expression. **E** KEGG map of differentially expressed proteins identified via proteomics analysis. Red represents the upregulated proteins in the P10 + PBL group. **F** Relative expression levels of the SUMO-1 and cytochrome C proteins. **G** Protein interactions between p53 and SUMO-1 were analyzed via co-IP. **H** The relative protein expression levels of PIAS1 and SENP1. **I** The localization of p53 in the P10 + PBL group was analyzed by fluorescence staining. Scale bar: 100 µm. Note: P10: hAMSCs were continuously expanded to the tenth passage in vitro; P10 + PBL: The P10 hAMSCs were cocultured with PBLs at a ratio of 1:100. The data are presented as the means ± SDs (*n* = 3). Statistical significance compared with the P10 group: ^#^*P* < 0.05, ^##^*P* < 0.01

Apoptotic bodies were observed via transmission electron microscopy in P10 hAMSCs treated with PBLs (Fig. 4A). To verify that the apoptotic cells in P10 hAMSCs were indeed aged cells, a β-galactosidase probe was employed to label the aged cells, while cleaved caspase 3 was used to label the apoptotic cells. The proportion of double-positive cells for β-galactosidase and cleaved-caspase 3 increased approximately threefold in the P10 + PBL group compared with the P10 group (Fig. 4B). The expression and ratio of mitochondrial apoptotic pathway-related markers, including Bax and Bcl-2, were increased in the P10 + PBL group, and immunofluorescence staining revealed a consistent trend (Fig. 4C, D). Further analysis via proteomic sequencing revealed that, compared with that in the P10 group, the protein expression of small ubiquitin-like modifier-1 (SUMO-1) in the P10 + PBL group was increased (Fig. 4E), and western blotting verified this result (Fig. 4F).

The activity and function of p53 are regulated by a variety of posttranslational modifications. Among these, the SUMOylation of p53 enhances its transcriptional activity and promotes its cytoplasmic localization, thereby facilitating the execution of apoptotic programs [36, 37]. Furthermore, the p53-dependent mitochondrial apoptotic pathway can stimulate the oligomerization of proapoptotic proteins and change the permeability of the mitochondrial outer membrane. Consequently, cytochrome C is released from the mitochondria into the cytoplasm, triggering a caspase cascade that ultimately activates caspase-3, the key executor of cell apoptosis, thereby inducing apoptosis [38]. In this study, consistent with the change in SUMO-1 protein expression, the expression of cytochrome C was significantly upregulated in the P10 + PBL group, as shown in Fig. 4F (*P* < 0.01). Furthermore, the co-IP results revealed that endogenous p53 bound more strongly to SUMO-1 after PBL treatment (Fig. 4G). SUMOylation is a dynamic and reversible process whose homeostasis is maintained by SUMOylation and de-SUMOylation enzymes [39]. PIAS1, as an E3-type SUMO ligase, can catalyze the SUMOylation of substrates, whereas sentrin-specific protease 1 (SENP1) mediates the de-SUMOylation of substrates. In this study, PBL treatment resulted in a significant increase in the expression level of the PIAS1 and SENP1 proteins (*P* < 0.01) (Fig. 4H), demonstrating that the loss of SUMOylation in P10 hAMSCs was reversed in the coculture system. Furthermore, dual fluorescein staining revealed that several p53 proteins translocated from the nucleus to the mitochondria in the P10 + PBL group (Fig. 4I). These data demonstrated that PBL reverses the dynamic equilibrium of SUMOylation modification in P10 hAMSCs to induce the apoptosis of aged cells through triggering the mitochondrial translocation of the p53 protein.

### PBLs enhanced the proliferation of younger MSCs via the activation of Serpinb2/NF-κB signaling

As previously mentioned, the anti-senescence effect of PBLs involved not only the induction of aged cell apoptosis but also the reversal of their growth arrest and the promotion of younger cell proliferation in P10 hAMSCs (Fig. 1C, F, and J) within the coculture system. The results in Fig. 4 demonstrated that PBLs induced apoptosis in aged P10 hAMSCs, resulting in a decrease in SA-β-Gal-positive cells in the coculture system. Here, we aimed to understand the mechanism underlying the remodeling of the proliferative potential of hAMSCs, with an increase in younger cells in the coculture system (Fig. 1C and F).

**Fig. 5:**
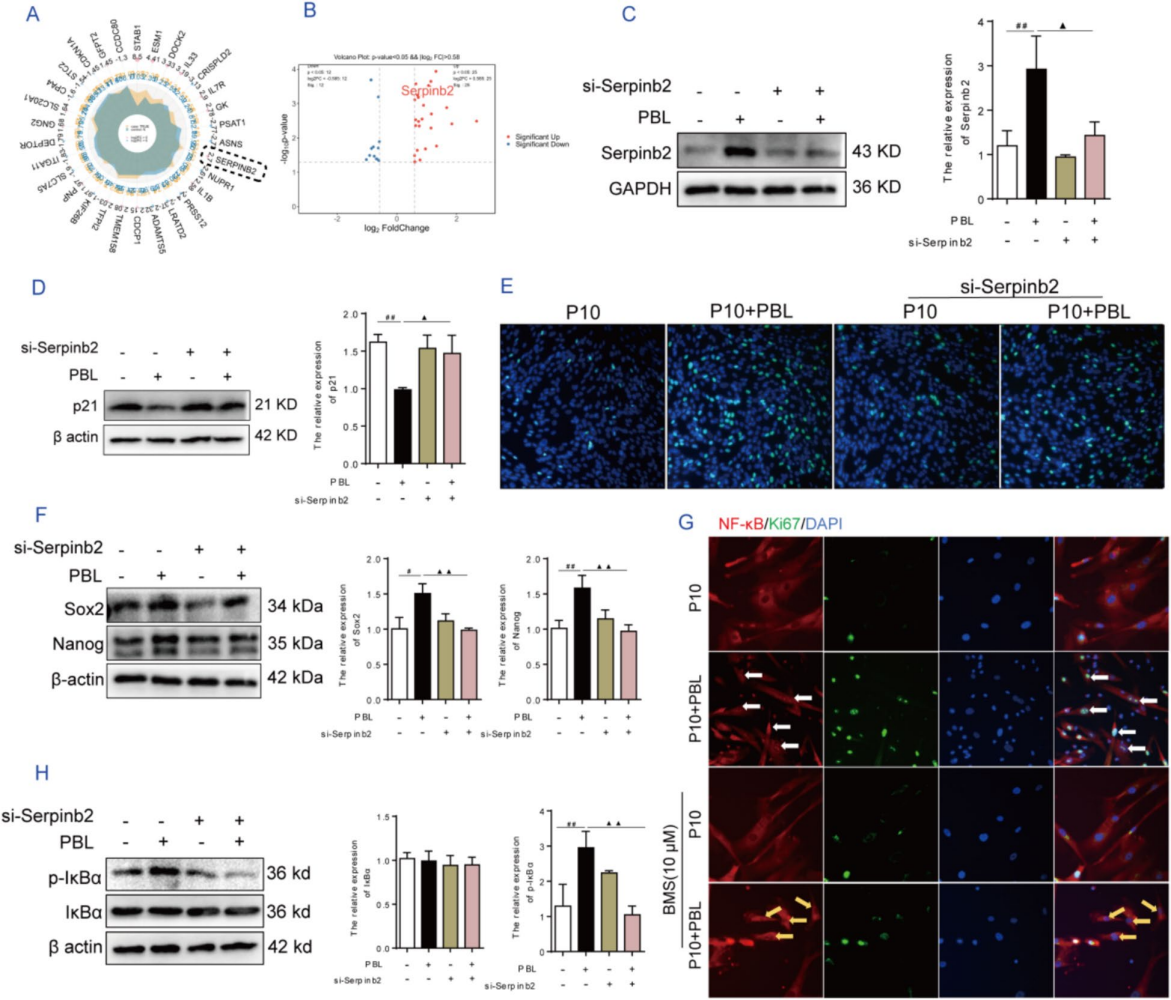
PBLs enhanced the proliferation of younger MSCs via the activation of Serpinb2/NF-κB signaling.** A** Differentially expressed genes with the smallest *p*-value or *q*-value in the transcriptome analysis of P10 vs P10 + PBL. **B** Volcano plot of differentially expressed proteins in the proteomic analysis of P10 vs P10 + PBL. **C** The relative expression level of the Serpinb2 protein in P10 hAMSCs. **D** The relative expression level of p21 protein in P10 hAMSCs. **E** The proliferation capacity of P10 hAMSCs was analyzed via EdU staining. **F** Relative protein expression levels of Sox2 and Nanog in P10 hAMSCs. **G** The localization and expression of NF-κB and Ki67 proteins were analyzed via immunofluorescence staining. **H** Relative protein expression level of IκB and phosphorylated IκB in P10 hAMSCs. Note: Scale bar: 100 µm. P10: hAMSCs were continuously expanded to the tenth passage in vitro; P10 + PBL: P10 hAMSCs were cocultured with PBLs at a ratio of 1:100. The data are presented as means ± SDs (*n* = 3). Statistical significance compared with the P10 group is indicated by ^#^*P* < 0.05; and ^##^*P* < 0.01; significance compared with the P10 + PBL group is indicated by ^▲^*P* < 0.01; and.^▲▲^*P* < 0.01

According to the RNA-seq data of P10 + PBL vs P10, *Serpinb2* was one of the 15 upregulated DEGs with the smallest *p*-value and *q*-value (Fig. 5A). Moreover, a proteomics analysis revealed that Serpinb2 was also upregulated (Fig. 5B). Serpinb2, also known as plasminogen activator inhibitor type 2 (PAI-2), is strongly upregulated in response to inflammatory stimulation [40]. TANK-binding kinase 1 induces Serpinb2 expression and activates NF-κB signaling [41], but the relationship between Serpinb2-induced expression and NF-κB signaling activation remains unclear. Consistent with the results of the proteomics analysis, Serpinb2 expression was upregulated after PBL treatment (Fig. 5C). However, *Serpinb2* knockdown resulted in the inability of PBL to reverse the senescence phenotype of P10 hAMSCs. Specifically, the protein expression of the senescence marker p21 (Fig. 5D), proliferative ability (Fig. 5E), and expression of stemness transcription factors such as Sox-2 and Nanog (Fig. 5F) in P10 hAMSCs remained unchanged in the *Serpinb2-*knockdown group with or without PBLs compared with those in the P10 group. These data suggest that *Serpinb2* plays a critical role in ameliorating senescence-associated phenotypes in PBL-treated P10 hAMSCs.

In addition, RNA-seq analysis indicated that NF-κB inflammatory signaling was the dominant cellular response induced by this coculture system in P10 hAMSCs (Fig. 3J). Therefore, we speculated that typical NF-κB prosurvival signaling might be involved in the protective effect of PBLs on the younger cell population of P10 hAMSCs. Our data revealed that PBLs activated the nuclear translocation of NF-κB in P10 hAMSCs within the coculture system, with a majority of NF-κB colocalizing with the proliferation marker Ki67 nuclear antigen (Additional file 1: Fig. S5). The inhibition of NF-κB nuclear translocation via the use of BMS-345541 (IKK inhibitor) resulted in a decreased Ki67 expression (Fig. 5G). However, *Serpinb2* knockdown significantly inhibited the phosphorylation of IκB in P10 hAMSCs induced by PBLs (Fig. 5H). Collectively, these data suggest that PBLs enhance younger MSC proliferation in P10 hAMSCs by promoting *Serpinb2* expression to activate the NF-κB signaling pathway in this coculture system.

### Enhanced therapeutic efficacy of rejuvenated hAMSCs obtained from the coculture system of PBLs and P10 hAMSCs

**Fig. 6:**
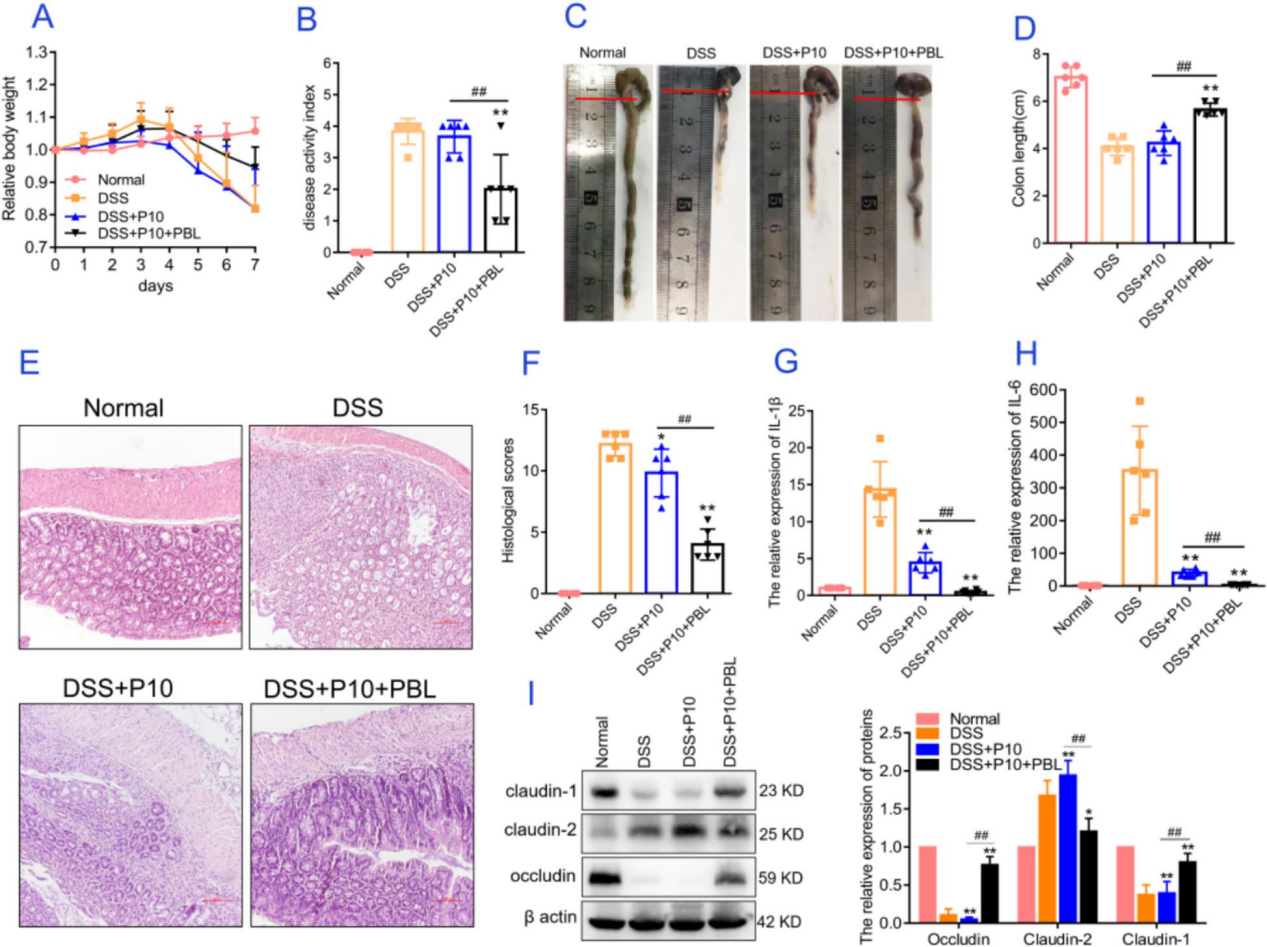
PBL preconditioning improved the therapeutic efficacy of P10 hAMSCs in experimental colitis model mice. Experimental procedure: Mice were subjected to DSS administration and injected via the tail vein with tenth passage hAMSCs or PBL-pretreated tenth passage hAMSCs (1 × 10^6^ cells) on DSS administration days 1 and 4. The normal and DSS groups received injections of equivalent volumes of PBS. **A** Body weights were recorded every day for 7 days. The relative change in body weight was calculated (*n* = 6). **B** The disease activity index (DAI) was evaluated on day 7 on the basis of symptoms (*n* = 6). **C** Colon length was measured on day 7 (*n* = 6). **D** Quantitative analysis of the data in Fig. 5C. **E** H&E staining was performed to analyze the histological changes in colons isolated on day 7. Scale bar: 100 µm. **F** H&E staining score. **G**, **H** Quantitative PCR analysis of the relative expression levels of IL-1β and IL-6 in the colon. **I** Relative expression levels of occludin, claudin-1, and claudin-2 in the colon. The data are presented as the means ± SDs. Note: Normal: normal control group; DSS: model group; DSS + P10: model group treated with P10 hAMSCs; DSS + P10 + PBL: model group treated with P10 hAMSCs pretreated with PBLs. Statistical significance compared with the DSS group is denoted as ^*^P < 0.05, ^**^P < 0.01. Compared with the DSS + P10 group is denoted as ^#^*P* < 0.05,.^##^*P* < 0.01

Transplanted MSCs can sense and respond to local inflammatory signals from the microenvironment, a process known as “MSC licensing.” Most preconditioning approaches enhance the efficacy of MSCs in vivo by initiating proinflammatory processes through cytokines or chemokines. To explore the efficacy of PBL-licensed MSCs in vivo, we used a dextran sodium sulfate (DSS)-induced experimental mouse colitis model. By the 5th day after DSS administration, the mice began to develop severe inflammatory colitis symptoms, including weight loss, loose stools, and bloody stools, with mortality peaking on the 7th day (Fig. 6A–D). Histological analysis revealed that DSS administration caused severe inflammation and damage to the colon mucosa (Fig. 6E and F). After tail vein injection of hAMSCs pretreated with PBLs, DSS-induced weight loss, the disease activity index (DAI), and colon length were significantly improved (*P* < 0.01). In contrast, P10 hAMSC treatment with only P10 hAMSCs did not alleviate these symptoms in the DSS-induced colitis model (Fig. 6A–D). Additionally, both hAMSC groups showed varying degrees of improvement in the histological score: the expression of proinflammation cytokines IL-1β and IL-6, and the expression of the tight junction proteins Occludin, Claudin-1, and Claudin-2. Notably, the group treated with hAMSCs pretreated with PBLs presented more pronounced regulatory effects (*P* < 0.01; Fig. 6E–I). Therefore, hAMSCs pretreated with PBLs demonstrated positive immunomodulatory ability and therapeutic efficacy in experimental colitis mice, significantly improving the inflammatory microenvironment and preserving intestinal barrier function. These findings highlight the potential of PBL-licensed hAMSCs as a promising therapeutic strategy for inflammatory bowel diseases.

### Rejuvenated hAMSCs obtained from the coculture system of PBLs and P10 hAMSCs presented a favorable safety profile

**Fig. 7:**
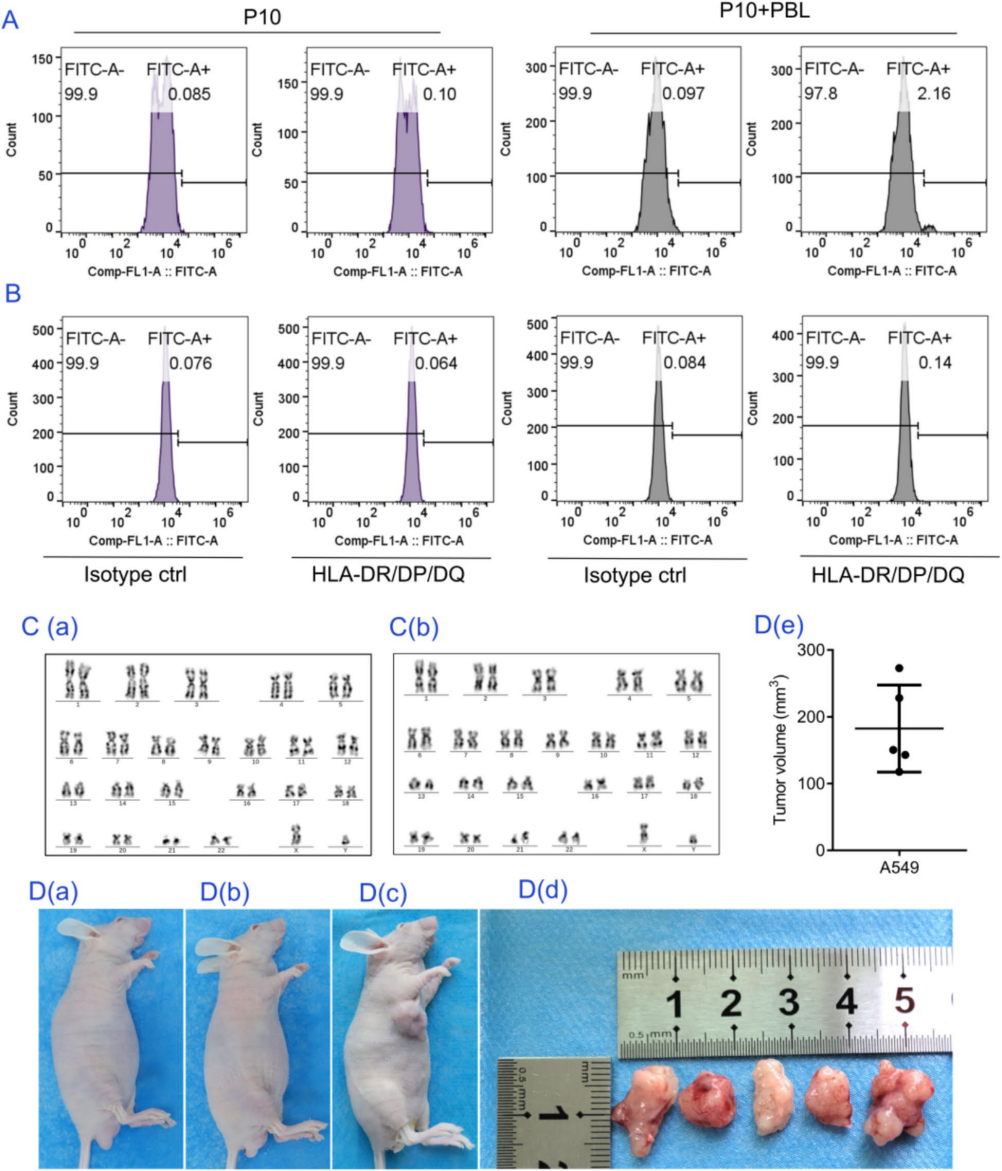
Safety evaluation of rejuvenated hAMSCs obtained from coculture of PBLs and P10 hAMSCs.** A**, **B** The number of HLA-DR/DP/DQ-positive cells in P10 hAMSCs was analyzed via flow cytometry: **A** PBLs were cocultured with P10 hAMSCs for 72 h, **B** PBLs were removed after 72 h of coculture, and P10 hAMSCs were analyzed after one subculture. **C** G-banding analysis of the karyotype of P10 hAMSCs cocultured with (a) or without (b) PBLs. **D** Tumorigenesis of hAMSCs was analyzed in nude mice: (a) P10 hAMSCs underarm inoculation, (b) P10 hAMSCs cocultured with PBLs, (c) A549 cells, (d) tumor tissue size in the A549 group, (e) tumor tissue volume statistics in the A549 group. Note: P10: hAMSCs were continuously expanded to the tenth passage in vitro; P10 + PBL: P10 hAMSCs were cocultured with PBLs at a ratio of 1:100. The data are expressed as the means ± SDs (*n* = 5)

For any stem cell-based therapy, safety is crucial, necessitating an investigation into the safety of hAMSCs rejuvenated with PBLs. Compared with MSCs, PBLs are derived from allogeneic sources and possess greater immunogenicity. If the two cell types fuse during crosstalk, leading to the expression of MHC class II molecules by rejuvenated hAMSCs, there is a risk of triggering graft-versus-host disease. Therefore, we analyzed the expression of classical MHC class II molecules in PBL-rejuvenated hAMSCs. PBLs were washed off after 72 h of coculture, and hAMSCs were collected for MHC II molecule analysis. The data revealed that the positive rate of HLA-DR/DP/DQ in the P10 + hAMSC group was 2.6%. However, considering that the number of PBLs was 100 times greater than the number of MSCs in this coculture system, it is likely that some PBLs were not completely removed. To eliminate this error, we subcultured MSCs rejuvenated by PBLs and then used them for further analysis. The data revealed that the expression of MHC II class molecules was negative in the subcultured MSCs (Fig. 7A and B). Additionally, G-banding data indicated that this coculture system did not change the karyotype of hAMSCs (Fig. 7C). Moreover, MSCs rejuvenated by PBLs were transplanted subcutaneously into nude mice, and no tumor mass formation was observed (Fig. 7D). These findings indicate a favorable safety profile of rejuvenated MSCs produced from our coculture system.

## Discussion

During prolonged ex vivo expansion of MSCs, cellular senescence poses a significant challenge in clinical applications. In this study, we found that allogeneic immune cells, particularly PBLs, effectively combated the hAMSC senescence in an in vitro coculture expansion system. PBLs not only induced the apoptosis of aged cells but also protected young cells against senescence and enhanced their stemness characteristics in P10 hAMSCs. This dual-action approach targeted both aged and young hAMSCs, utilizing distinct mechanisms to improve cell function, highlighting the effectiveness of PBLs in rejuvenating P10 hAMSCs.

In aged hAMSCs, allogeneic PBLs are activated by MSCs, causing a significant alteration in their secretion profile (Fig. 2). Although the increased inflammatory cytokines did not perfectly reverse the senescence phenotype of MSCs, they did contribute to promoting cell division. Several studies have reported the important contribution of inflammatory factors to tissue repair and regeneration [42, 43]. Accordingly, these increased cytokine levels provide indirect evidence for the activation of PBLs by MSCs. Previous studies have shown that senescent cells can activate autologous immune cells, resulting in immune clearance [27, 28, 44]. The exact reasons why allogeneic PBLs target apoptosis in aged cells but not in young cells are as follows. First, aged cells produce and secrete SASP factors, effectively recruiting NK and T cells for their elimination [45, 46]. Second, aged cells release danger signal molecules and senescence-associated antigen peptides via enhanced MHC class I molecules, making them uniquely immunogenic and capable of efficiently activating CD8 + T cells [6, 47]. In addition, this study revealed a new perspective on how inflammation restructures the dynamic equilibrium of SUMOylation in aging MSCs. In the coculture system, SUMOylation of p53 induced mitochondrial translocation and drove apoptotic events.

MSCs are known to constitute a heterogeneous population with varying proliferative, pluripotent, and immunomodulatory capacities [6, 18]. The method described in this study represents a two-pronged approach to combat hAMSC senescence by controlling the heterogeneity of long-term in vitro hAMSC expansion. PBLs induced the apoptosis of aged MSCs while concurrently enhancing the stemness characteristics of nonaged MSCs. Therefore, this study differs from senolytic drug treatments known as senolytics, which aim to clear senescent cells [19, 22, 48]. Despite the effectiveness of PBLs, a minute fraction of senescent P10 hAMSCs survived following PBL treatment (Fig. 1F). In vivo, senescent cell accumulation may indicate immune evasion and the failure of endogenous immune monitoring mechanisms. For instance, a decrease in the number and activity of immune cells leads to the slow or even incomplete elimination of senescent cells. Alternatively, changes in the expression of MHC molecules [49] and high expression of immunosuppressive ligands such as PD-L1 [50] may allow senescent cells to evade immune system recognition. Therefore, even with a high proportion of PBLs, some aged cells may not be cleared due to unknown mechanisms.

Cellular senescence is a continuous and organized process characterized by irreversible cell cycle arrest and apoptosis resistance. During long-term in vitro expansion, significant alterations in gene expression patterns occur between early- and late-generation MSCs, particularly in the cell cycle profile, indicating the aging of the cultures [47]. In our study, we found that PBLs not only induced apoptosis in aged cells but also remodeled the gene expression patterns and biological properties of P10 hAMSCs, akin to cell remodeling processes [51]. This resulted in a significant decrease in the expression of the cell cycle negative regulatory factors P21 and P16 (Fig. 1G). Consequently, the proportion of P10 hAMSCs in the S-phase and the ratio of EdU-positive cells were substantially increased (Fig. 1J and C), indicating that the cell cycle or proliferation arrest was reversed. Furthermore, PBLs effectively improved the relative length of telomeres, as well as their colony formation ability and differentiation potential (Fig. 1I, K–M). Therefore, PBLs rejuvenated hAMSCs by targeting nonaged cells in the P10 hAMSC population, restoring their clinical application advantages. Despite analyzing changes in the typical prosurvival signaling pathway NF-κB in the MSCs in this coculture system, it remains uncertain whether this process confers inflammatory memory to hAMSCs, as observed in skin stem cells [52] and hair follicle stem cells [53], enabling a rapid response to subsequent danger. Additionally, the coculture system extended telomere length while promoting the expansion of young hAMSCs, posing an intriguing scientific question worth exploring. Therefore, telomere damage repair, telomerase activity, and telomere transmission [54] may be involved in the coculture system.

Additionally, although this study speculated that PBLs enhance the stemness of relatively young hAMSCs, and that most aged cells underwent apoptosis after PBL treatment in P10 hAMSCs (Fig. 4), this study cannot completely conclude that PBLs do not stimulate aged P10 hAMSCs to enter a state of rereplication because of the heterogeneity of senescent cells [55]. While the cell cycle arrest of senescent cells is traditionally considered irreversible, recent research suggests that these cells may re-enter the cell cycle if they acquire epigenetic changes that inhibit the expression of antiaging genes [56]. Hence, there is a chance of senescent cells reentering the tumor cell cycle [57] or being reprogrammed into pluripotent stem cells [58].

Moreover, the rejuvenated MSCs demonstrated promising therapeutic efficacy. The method proposed in this study could be applied in clinical settings by collecting patients' own peripheral blood, thereby avoiding issues such as tumorigenesis, immune rejection, high costs, and poor effectiveness associated with other approaches. This makes it a promising strategy to address stem cell senescence.

Furthermore, aside from maintaining a youthful state for clinical-grade cell populations, MSCs rejuvenated by this coculture system may offer another potential advantage in treating immune disorders. Many preconditioning methods increase MSC treatment success by initiating proinflammatory processes with cytokines/chemokines or growth factors. For example, IFN-γ stimulation increases the secretion of immunomodulators, including PGE2, HGF, TGF-β, and MCP-1 [31]. TNF-α stimulation enhances the immunosuppressive, homing, and tissue repair abilities of MSCs [59]. IL-1β stimulation regulates the immune balance of organisms by upregulating TGF-β1 and MMPs, promoting MSC migration and wound healing, and improving the efficacy of MSCs for various purposes [60]. Therefore, PBLs, comprising T, B, and NK cells, could enhance the effectiveness of MSCs in immune disorders such as osteoarthritis, wound healing, and diabetes because of their stimulating and activating effects on MSCs. Although specific quantification of cells or components in PBLs has not yet been achieved, the source of PBLs is not limited to the donor, and even PBLs isolated from older individuals could effectively rejuvenate MSCs in this study (Additional file 1: Fig. S1).

## Conclusions

In conclusion, this coculture system offers a novel approach that effectively rejuvenates MSCs and addresses cellular senescence during long-term in vitro expansion of MSCs, achieving a “two birds with one stone” solution. This approach not only facilitates the prompt clearance of senescent cells but also allows for the maintenance and even enhancement of cellular stemness and biological functions. Here PBLs present a dual-action mechanism to rejuvenate MSCs through targeting senescent cell clearance while promoting the proliferation of younger cells.

## Methods

### Isolation, culture, and identification of hAMSCs

The use of human amniotic membranes was approved by the Ethics Committee of Zunyi Medical University (Zunyi, China). Human amniotic membrane tissues were collected from the placental amniotic tissues of healthy donors undergoing cesarean delivery at term, following the acquisition of written informed consent from the donors or their legal relatives. The procedures for isolating, culturing, and identifying hAMSCs adhered to those described in our previously published studies [22, 23]. Briefly, under sterile conditions, the amniotic membrane was mechanically separated and repeatedly washed with Dulbecco’s Phosphate-Buffered Saline (D-PBS) containing 1% penicillin–streptomycin (PS) to remove residual blood. The membrane was then minced into 1–2 cm^2^ fragments and collected in 50 mL centrifuge tubes. A double volume of 0.05% trypsin solution was added, followed by shaking digestion at 37 °C for 40 min. After washing the remaining tissue fragments, an equal volume of the digestion solution (0.5 mg/mL collagenase II with 0.05 mg/mL DNase I) was added for shaking digestion at 37 °C for 1.5 h. hAMSCs were obtained following filtration and centrifugation. The cells were resuspended in freshly prepared low-glucose (LG)-DMEM/F12 complete medium and seeded into sterile T25 flasks at a density of 5 × 10^5^ cells/flask. Upon reaching 80% confluency, the cells were passaged into new T25 flasks (designated as Passage 1, P1).

For hAMSC characterization: immunocytochemical staining confirmed positive expression of vimentin and negative expression of keratin in the cells. Additionally, flow cytometry analysis demonstrated positive expression of characteristic MSC surface markers (CD29, CD105, CD73, CD90, and CD44), and negative expression of hematopoietic stem cell markers (CD34, CD11b, CD45, and HLA-DR). Cells from passages 4 (P4) and 10 (P10) were utilized for subsequent experiments.

### Isolation of human PBLs

Human peripheral blood mononuclear cells (PBMCs) were isolated via Ficoll–Hypaque density gradient centrifugation [61]. Briefly, 4 mL of Histopaque®−1077 was added to a 15-mL centrifuge tube, followed by the addition of an equal volume of diluted normal peripheral blood along the tube wall. The mixture was centrifuged at 700 × *g* for 20 min at 18–22 °C, and the middle white layer was aspirated. An equal volume of sterile D-PBS was added to the blood, which was subsequently centrifuged at 300 × *g* for 10 min at 18–22 °C. The pellet was washed once with sterile D-PBS, and the supernatant was discarded. The pellet was then counted and suspended in LG-DMEM containing 10% (v/v) FBS, penicillin, and streptomycin (1%), and 10 ng/mL human bFGF and maintained at 37 °C in an incubator with an atmosphere consisting of 5% CO_2_, 95% air, and 100% relative humidity. After approximately 2 h, peripheral blood monocytes (PBMs) adhered to the bottom of the cell culture plate, while nonadherent cells were identified as PBLs [62].

### Coculture settings in young hAMSCs

After freshly isolated PBMCs were cocultured with P4 hAMSCs for 48 h at an effector-to-target ratio of 100:1 in LG-DMEM containing 10% (v/v) FBS, penicillin, and streptomycin (1%), and 10 ng/mL human bFGF, the hAMSCs were harvested for subsequent analysis.

### Coculture settings in aged hAMSCs

Freshly isolated PBMCs and their subsets (PBLs and PBMs) were cocultured with P10 hAMSCs at a 100:1 ratio for 72 h in LG-DMEM containing 10% (v/v) FBS, penicillin, and streptomycin (1%), and 10 ng/mL human bFGF. The hAMSCs were then harvested for further analysis.

### EdU assay

The proliferation potential of hAMSCs was assessed via the cell-light EdU Apollo 488 in vitro kit (C10310-3; RiboBio, Guangzhou, China) following the manufacturer’s instructions. hAMSCs were incubated with fresh medium containing 50 μM EdU for 2 h. The medium was then discarded, and the cells were washed with D-PBS. Next, the cells were fixed with 4% paraformaldehyde for 30 min at 20–28 °C, followed by incubation with 2 mg/mL glycine for 5 min to neutralize excess aldehyde groups. After being washed with D-PBS, the cells were treated with 0.25% Triton X-100 (v/v) for 10 min and then incubated with Apollo staining solution for 30 min in darkness. Following another wash with D-PBS, the nuclei were stained with Hoechst 33,342 and observed under an ECLIPSE Ti fluorescence microscope (Nikon, Japan).

### β-galactosidase staining

The morphology of senescent cells was observed and quantified via a senescence-associated β-galactosidase (SA-β-Gal) staining kit (C0602; Beyotime, Shanghai, China) following the manufacturer’s instructions and as described previously [22, 23]. Briefly, hAMSCs were washed with D-PBS, fixed with a fixing solution for 30 min at 20–28 °C, and then treated with a freshly prepared β-galactosidase staining solution at 37 °C in darkness for 4 h. The staining was stopped, and the cells were observed under an optical microscope (ECLIPSE Ti; Nikon, Tokyo, Japan).

### Western blot analysis

The relative expression of individual proteins was assessed via western blot analysis. Total cellular protein was extracted using RIPA lysis buffer (R0010; Solarbio, Beijing, China). Nuclear and cytoplasmic proteins were extracted following the instructions of NE-PER™ Nuclear and Cytoplasmic Extraction Reagents (78,833; Thermo Fisher Scientific, USA). The protein concentrations were determined using a BCA Kit (PC0020; Solarbio, Beijing, China) following the manufacturer’s instructions. The denatured protein samples were subsequently loaded onto 10% or 12.5% SDS–PAGE gels for separation. Primary antibodies (listed in Additional file 3: Table S1) were added to the membranes, which were subsequently incubated overnight at 4 °C. After washing with TBST to remove unbound antibodies, the membranes were exposed to horseradish peroxidase (HRP)-labeled goat anti-rabbit IgG and goat anti-mouse IgG for 1.5 h at 20–28 °C. Following additional washing steps with TBST, the membranes were treated with enhanced chemiluminescence (ECL) solution (abs9434; Absin, Shanghai, China). Finally, the membranes were exposed using the Bio-Rad Chemi Doc™ MP Imaging System darkroom (Bio-Rad, Hercules, CA, USA), and images were recorded. ImageJ 1.46a software (NIH, Bethesda, MD, USA) was used to analyze the protein bands. β-actin was used as the internal reference for error correction.

### ROS assessment

The levels of cellular ROS in P10 hAMSCs, both cocultured with and without PBLs, were analyzed following the protocol of the ROS assay kit (CA1410; Solarbio, Beijing, China). Briefly, hAMSCs were incubated with serum-free dilute DCFH-DA working solution (10 μmol/L) at 37 °C for 20 min. Next, the cells were washed three times with serum-free cell culture medium to remove any residual DCFH-DA outside the cells. Finally, ROS expression in the cells was observed under an ECLIPSE Ti fluorescence microscope (Nikon, Japan).

### Relative telomere length assay for hAMSCs

P10 hAMSCs cocultured with or without PBLs were harvested, and genomic DNA was extracted. The relative telomere length of hAMSCs was then measured using the Human Telomere Length Quantification qPCR Assay Kit (EQ022; ELK Biotechnology, Wuhan, China) following the manufacturer’s protocol.

### Cell cycle analysis

P10 hAMSCs cocultured with or without PBLs were harvested and analyzed according to the instructions of a DNA content detection Kit (CA1510; Solarbio, China). Briefly, the cells were collected and fixed with 500 μL of 70% precooled ethanol for 2 h. The cells were then washed with D-PBS and incubated with 100 μL of RNase A at 37 °C for 30 min. After incubation with 400 μL of PI staining solution at 4 °C for 30 min in darkness, the cells were analyzed via an Accuri™ C6 Plus flow cytometer (BD, Franklin Lakes, NJ, USA).

### Colony formation assay

P10 hAMSCs were seeded at a density of 1,000 cells in a 10-cm diameter cell culture dish. After 14 h, the cells were cocultured with PBLs in a cell-to-cell manner, with medium replenished every 4 days. On the 15th day, the cells were removed and fixed in 4% paraformaldehyde for 30 min at 20–28 °C. After being washed with D-PBS, the cells were stained with 1% crystal violet for 30 min. Subsequently, the cells were washed with D-PBS, dried, and observed under a camera (Galaxy S21; Samsung, Korea).

### Trilineage differentiation assay

P10 hAMSCs were seeded in a 6-well cell culture plate at a density of 2 × 10^5^ cells per well, followed by the addition of PBLs after 14 h. Upon reaching 100%, 70%, and 80% confluence, the culture medium was changed to adipogenic induction medium (HUXUC-90031; Cyagen, Guangzhou, China), osteogenic induction medium (HUXUC-90021; Cyagen, Guangzhou, China), or chondrocyte induction medium (HUXUC-90042; Cyagen, Guangzhou, China), respectively, according to the manufacturer's instructions. The medium was replaced with fresh medium twice a week throughout the differentiation induction period. On the 18th day, hAMSCs that differentiated into adipocytes were visualized via Oil Red O staining, which revealed accumulated lipid vacuoles. Similarly, on the 21 st day, alizarin red S staining was performed to detect the formation of calcium nodules indicative of hAMSC osteogenic differentiation. In addition, toluidine blue staining was performed to assess the chondrocyte differentiation of hAMSCs.

### PBL proliferation assay

The proliferation of PBLs cocultured with or without P10 hAMSCs was detected via flow cytometry, using the CFDA, SE Cell Proliferation and Tracer Assay Kit (CA1200; Solarbio, Beijing, China) according to the manufacturer’s instructions. Briefly, freshly isolated PBLs were counted and incubated with CFDA or SE working solution in a cell incubator at 37 °C in darkness for 10 min, followed by washing with D-PBS and centrifugation at 200 × *g* to collect the cells. The labeled PBLs were then cocultured with or without P10 hAMSCs for 12 h. Finally, the cells were collected via flow cytometry, and the mean fluorescence intensity was measured. Decreased fluorescence intensity indicated increased cell division.

### Transcriptome analysis of PBLs

PBLs cocultured with or without P10 hAMSCs for 12 h were harvested and treated with 1 mL of TRIzol. The cells were then transported on dry ice to Suzhou Panomik Biotechnology Company (Suzhou, China) for transcriptome sequencing analysis and data processing. Briefly, total RNA was extracted from cells using TRIzol reagent, and RNA integrity was assessed using the Agilent 2100 Bioanalyzer (Agilent Technologies, Santa Clara, CA, USA). Poly(A)-enriched mRNA was isolated from total RNA for library construction. Sequencing libraries were prepared and subjected to paired-end sequencing (2 × 150 bp) on an Illumina platform. Raw sequencing data were filtered to obtain clean reads, which were then aligned to the human reference genome (GRCh38/hg38). Gene expression levels were quantified based on the alignment results. Differential expression analysis, functional enrichment analysis, and hierarchical clustering were subsequently performed across sample groups.

### Transcriptome analysis of hAMSCs

P10 hAMSCs cocultured with or without PBLs were harvested and treated with 1 mL of TRIzol. The cells were then transported on dry ice to Shanghai OE Biotech Co., Ltd (Shanghai, China) for transcriptome sequencing analysis and data processing. The experimental procedure was described in the above transcriptome analysis of PBLs.

### Proteome analysis of hAMSCs

P10 hAMSCs cocultured with or without PBL were harvested and snap-frozen in liquid nitrogen. The cells were then transported on dry ice to Suzhou Panomik Biotechnology Company (Suzhou, China) for quantitative proteomics analysis and data processing. Briefly, total protein was extracted and quantified, followed by equal aliquoting. Proteins were then reduced and alkylated to cleave disulfide bonds and block free sulfhydryl groups. Subsequently, tryptic digestion was performed to generate peptides. The resulting peptides were incubated with iTRAQ reagents, in which isobaric tags were covalently conjugated to peptide N-termini via amine-specific reactions. Equally pooled iTRAQ-labeled peptides from different samples underwent pre-fractionation using high-pH reverse-phase chromatography to reduce sample complexity. The fractionated peptides were separated by nanoflow liquid chromatography (nanoLC) and analyzed by tandem mass spectrometry (MS/MS). During MS1 scans, iTRAQ-tagged peptides exhibited identical mass-to-charge (m/z) ratios; conversely, in MS2 fragmentation, reporter ions (m/z 114–121) were released for quantification. Protein identification and relative quantification were achieved through database searching using MaxQuant software, which calculated reporter ion intensity ratios. Finally, differential expression analysis, functional enrichment analysis, and clustering analysis were performed on the samples.

### Transmission electron microscope

P10 hAMSCs cocultured with or without PBLs were harvested and analyzed with a transmission electron microscope (HITACHI, Japan, H7650). Briefly, hAMSCs were harvested by trypsin digestion, washed once with D-PBS, and subsequently fixed with a glutaraldehyde fixative solution. The next day, the fixed cells were sent to the electron microscope room of Zunyi Medical University for sample preparation, staining, sectioning, and observation.

### Double staining analysis of senescent and apoptotic cells

P10 hAMSCs, which were cocultured with or without PBLs for 12 h, were collected for analysis of senescent and apoptotic cells via β-galactosidase and active caspase 3 as markers, respectively. Cell proportions were determined by flow cytometry following cell labeling as per the manufacturer’s instructions. Senescent cells were labeled using the CellEvent™ Senescence Green Flow Cytometry Assay Kit (C10841; Invitrogen, USA). Briefly, the cells were fixed with 4% paraformaldehyde for 10 min at 20–28 °C, washed with 1% BSA, and incubated with a freshly prepared CellEvent™ Senescence Green Probe working solution at 37 °C in the dark for 1.5 h. The cells were subsequently washed, permeabilized with 0.25% (v/v) Triton X-100, suspended in 1% BSA, and incubated with PE-conjugated rabbit anti-active Caspase-3 (570,184; BD, USA) at 2–8 °C for 30 min in the dark. Following the removal of the residual antibody solution, the cells were washed and analyzed by flow cytometry.

### Immunofluorescence analysis

For staining, P10 hAMSCs cocultured with or without PBLs were harvested. After washing with PBS, cells were permeabilized with 0.3% Triton X-100 in PBS for 10 min at 24–28 °C. Following PBS washes, antigens were blocked with 2% BSA at 24–28 °C. For immunofluorescence staining, cells were incubated with primary antibodies against Bax (Huabio, EM1203, 1:200) and Bcl-2 (Huabio, ET1603-11, 1:100) at 4 °C overnight. After PBS rinsing, cells were incubated with FITC-conjugated goat anti-mouse and PE-conjugated goat anti-rabbit secondary antibodies for 1 h at 24–28 °C. Nuclei were counterstained with 4′,6-diamidino-2-phenylindole (DAPI). Stained cells were examined under a fluorescence microscope (IX-71, Olympus, Tokyo, Japan).

### Assessment of ubiquitin-like modification of p53 protein

The co-immunoprecipitation was used to analyze the ubiquitin-like modification of p53 protein. P10 hAMSCs cocultured with or without PBLs were harvested for analysis. The procedure was as follows: (1) Cells were gently scraped from the culture dish using a cell scraper, collected by low-speed centrifugation, and washed twice with pre-cooled PBS. After adding 1 mL RIPA lysis buffer, the cells were pipetted repeatedly and placed on ice for 10–20 min for complete lysis. The lysate was sonicated using an ultrasonic disruptor until transparent and non-viscous. Following 30-min incubation on ice, the lysate was centrifuged at 12,000 rpm and 4 °C for 10 min. The supernatant was collected and stored at − 80 °C; (2) Column loading and incubation: The total antibody (ab26, 1:100) volume was adjusted to 500 μL with 1 × PBS. To 40 μL magnetic bead suspension (Dai-an, M0134), 500 μL 1 × PBS was added, mixed thoroughly, and magnetically separated. This washing step was repeated twice. The diluted antibody was added to pre-washed magnetic beads, gently mixed, and incubated on a shaker at room temperature for 30 min. After magnetic separation, 500 μL 1 × PBS was used for four washes; (3) Antigen–antibody-bead complex formation: 400 μL prepared antigen sample was added to the magnetic beads, gently mixed, and incubated overnight at 4 °C with shaking. Post magnetic separation, the supernatant was discarded and washed four times with 1 × PBST; (4) Antigen elution: Magnetic beads were separated, resuspended in 20 μL 2 × protein loading buffer, mixed well, and boiled at 95 °C for 5 min. After magnetic separation, the supernatant was collected for SDS-PAGE analysis.

### Mitochondria and p53 co-staining analysis

P10 hAMSCs cocultured with or without PBLs were harvested. The cells were added to pre-warmed MitoTracker® Red CMXRos staining solution (Solarbio, M9940) at a final concentration of 100 nM, then incubated at 37 °C for 30 min. After washing twice with PBS, p53 staining was performed according to standard immunofluorescence protocols. Notably, the primary antibody used was rabbit anti-p53 (Abcam, ab32049, 1:100), and the secondary antibody was FITC-conjugated goat anti-rabbit. Finally, the stained cells were examined under a fluorescence microscope (IX-71, Olympus, Tokyo, Japan).

### Serpinb2-targeting siRNA design and validation

Small interfering RNAs (siRNAs) targeting human *Serpinb2* gene (NCBI accession: NM_001143818.2) was designed and three independent siRNA sequences were synthesized by Sangon (Shanghai, China) with the following sequences: siRNA#1: si-S-5′-GCAGAUCCAGAAGGGUAGUUATT, si-A-5′-UAACUACCCUUCUGGAU CUGCTT; siRNA#2: si-S-5′-GCUUUAUCCUUUCCGUGUAAATT, si-A-5′-UUUA CACGGAAAGGAUAAAGCTT; siRNA#3: si-S-5′-CCACAGUUUGUGGCAGAU CAUTT, si-A-5′-AUGAUCUGCCACAAACU GUGGTT. For RNA interference and transfection, P10 hAMSCs were seeded in 6-well plates at a density of 2 × 10^5^ cells/well and allowed to adhere for 24 h. siRNA (50 nM) and Lipofectamine 2000 reagent (Invitrogen) were diluted in Opti-MEM medium (Gibco) according to the manufacturer’s protocol, followed by incubation at ambient temperature for 15 min. The siRNA-Lipofectamine complexes were then added to the cells. After 24 h of transfection, the medium was replaced with fresh complete medium. The hAMSCs were harvested after 72 h to detect relevant indicators.

### Intravenous injection of hAMSCs in the experimental colitis model

To investigate the immunomodulatory capability and in vivo efficacy of P10 hAMSCs following PBL pretreatment, we induced an experimental colitis model in mice via DSS. The animal experimental protocols were reviewed and approved by the Animal Experiment Ethics Committee of Zunyi Medical University (license number: Lunshen [2020] 2–468). Six- to eight-week-old C57BL/6J mice were randomly divided into four groups (*n* = 6): normal, DSS, DSS + P10, and DSS + P10 + PBL. Except for the normal group, all the mice received 2.5% (w/v) DSS (Millipore, Billerica, MA, USA) in their drinking water. The mice in the normal group received pure water. On the first and fourth days of DSS administration, hAMSCs (1 × 10^6^) were injected via the tail vein in the P10 and P10 + PBL groups via P10- and PBL-pretreated P10 hAMSCs, respectively. The mice in the normal and DSS groups received an equivalent volume of sterile PBS. Throughout the observation period, we recorded the body weight at a specified time daily and monitored for the presence of loose and bloody stools. The DAI was calculated according to changes in body weight, diarrhea, hematochezia, and mortality [63]. Seven days after hAMSC injection, the mice were euthanized via isoflurane gas inhalation, and the intact colon length was measured. Pathological analysis was conducted by fixing colon segments overnight in 4% paraformaldehyde at 4 °C, embedding them in paraffin, and sectioning them into 4 μm slices for H&E staining. Inflammatory cell infiltration and the intact structure of crypt and goblet cells in the colon were observed under a microscope (Eclipse Ti, Nikon, Japan). Histopathological scoring was performed according to a previously reported method [64].

### HLA-DR/DP/DQ analysis

The cell suspensions were incubated with 5 μL of FITC-conjugated anti-human HLA-DR, DP, or DQ antibodies (361,705; Biolegend, USA) at 2–8 °C for 30 min, washed with D-PBS to remove excess antibody, and then analyzed via flow cytometry. An isotype control was used for each sample.

### G-banding analysis of hAMSC karyotypes

hAMSCs cocultured with or without PBLs were treated with colchicine (0.25 μg/mL) for 3 h. The cells were then collected and treated with KCl (0.075 mol/L) at 37 °C for 8 min. After centrifugation (300 × *g*, 5 min) and fixation with a fixing solution (glacial acetic acid:methanol = 1:3) at 37 °C for 5 min, the cells were suspended again in an appropriate fixative and sectioned. The sections were stained with Giemsa and analyzed for G-banding analysis (Leica GSL 120; Germany).

### Tumorigenicity analysis of hAMSCs in nude mice

Fifteen 4-week-old nude mice were obtained from Spiff Beijing Biotechnology Co., Ltd (SCXK 2019–0010). The animal experimental protocols were reviewed and approved by the Animal Experiment Ethics Committee of Zunyi Medical University (license number: Lunshen[2020]2–468). After 1 week of adaptive feeding, the mice were divided into the following groups: P10 group (injected with 10^7^ P10 hAMSCs into the right axilla; *n* = 5), P10 + PBL group (injected with 10^7^ P10 hAMSCs after PBL was cocultured into the right axilla; *n* = 5), and A549 group (inoculated with 10^7^ A549 cells in the axilla; *n* = 5). Tumor formation and growth were monitored daily postinjection. The mice in the A549 group were euthanized one month after cell inoculation, and the tumor tissue volume was recorded. The remaining nude mice were euthanized 3 months after inoculation.

### Quantification and statistical analysis

The data are expressed as the means ± standard errors and were obtained from a minimum of three independent experiments. Statistical analysis was performed via SPSS software (version 13.0; IBM Corp, Chicago, USA). Differences between groups were assessed via one-way ANOVA and/or Student’s *t* test. *P* values less than 0.05 were considered statistically significant.

## Supplementary Information

Additional file 1: Fig. S1. The proproliferation and anti-senescence effects of PBMCs and PBLs were analyzed in the coculture system. Fig. S2. Coculture with hAMSCs activated the secretion profile of PBLs. Fig. S3. Effects of cytokines on hAMSC phenotype and characteristics. Fig. S4. KEGG enrichment analysis of the RNA-seq data. Fig. S5. Expression of NF-κB in P10 hAMSCs.

Additional file 2: Video S1. Real-time monitoring of P10-hAMSC growth for 72 h in culture plate. Video S2. Real-time monitoring of P10-hAMSCs co-cultured with PBLs in culture plate over a 72-h period.

Additional file 3: Table S1. Key resource information used in this study.

Additional file 4.

## Data Availability

The raw sequence data of RNA-seq reported in this paper have been deposited in the Gene Expression Omnibus database of NCBI and can be publicly accessible through accession numbers GSE307002 [65] and GSE307003 [66]. The mass spectrometry proteomics data have been deposited to the ProteomeXchange Consortium (https://proteomecentral.proteomexchange.org) via the iProX partner repository with the dataset identifier PXD066147 [67].

## Abbreviations

hAMSCs: Human amniotic mesenchymal stem cells
MSCs: Mesenchymal stem cells
PBLs: Peripheral blood lymphocytes
PBMCs: Peripheral blood mononuclear cells
PBMs: Peripheral blood monocytes
SASP: Senescence-associated secretory phenotype
NK: Natural killer
SA-β-gal: Senescence-associated β-galactosidase
ROS: Reactive oxygen species
hU-MSCs: Human umbilical cord mesenchymal stem cells
UCB-MSCs: Human umbilical cord blood mesenchymal stem cells
SUMO-1: Small ubiquitin-like modifier-1
SENP1: Sentrin-specific protease 1
PIAS1: E3 SUMO-protein ligase PIAS1
PAI2: Plasminogen activator inhibitor type 2
DSS: Dextran sodium sulfate
DAI: Disease activity index
