# Supplementary material for: Rejuvenation of mesenchymal stem cells by human peripheral blood lymphocytes
Source: BMC Biol. 2025 Nov 25;23:370. doi: 10.1186/s12915-025-02472-9 (PMC12750958; doi:10.1186/s12915-025-02472-9)
Supplement: Supplementary file 1 — Additional file 1: Fig. S1. The proproliferation and anti-senescence effects of PBMCs and PBLs were analyzed in the coculture system. Fig. S2. Coculture with hAMSCs activated the secretion profile of PBLs. Fig. S3. Effects of cytokines on hAMSC phenotype and characteristics. Fig. S4. KEGG enrichment analysis of the RNA-seq data. Fig. S5. Expression of NF-κB in P10 hAMSCs. [file 12915_2025_2472_MOESM1_ESM.docx]

### **Rejuvenation of mesenchymal stem cells by human peripheral blood lymphocytes: A dual-mechanism for targeting senescent cell clearance and promoting cell proliferation in a coculture system**

Yi Luo ^1,2^, Xin-Xin Zhu ^1,4^, Qing-Rong Le ^1,4^, Wen-Ting Chen ^1,4^, Yan Xu ^1,4^, Xue-Mei Chen ^1,5^, Huan Yuan ^1,4^, Xu Yang ^1,4^, Jun-Wei Xu ^2*^, Jian-Jiang Zhong ^1,3*^, Jian-Hui Xiao^1,4,5,6*^

^1^ Institute of Medicinal Biotechnology & Center for Translational Medicine, Affiliated Hospital of Zunyi Medical University, 149 Dalian Road, Huichuan District, Zunyi 563003, China.

^2^ Faculty of Life Science and Technology, Kunming University of Science and Technology, Kunming, 650500, China.

^3^ State Key Laboratory of Microbial Metabolism, and School of Life Sciences & Biotechnology, Shanghai Jiao Tong University, 800 Dongchuan Road, Shanghai 200240, China.

^4^ Guizhou Provincial Key Laboratory of Medicinal Biotechnology & Research Center for Translational Medicine in Colleges and Universities, Affiliated Hospital of Zunyi Medical University, 149 Dalian Road, Huichuan District, Zunyi 563003, China.

^5^ Department of Pediatrics, Affiliated Hospital of Zunyi Medical University, 149 Dalian Road, Huichuan District, Zunyi 563003, China

^6^ Lead contact

^*^Corresponding authors: jwxu@kust.edu.cn (J.-W. Xu); jjzhong@sjtu.edu.cn (J.-J. Zhong); jhxiao@zmu.edu.cn (J.-H. Xiao)

**Supplementary Figures**


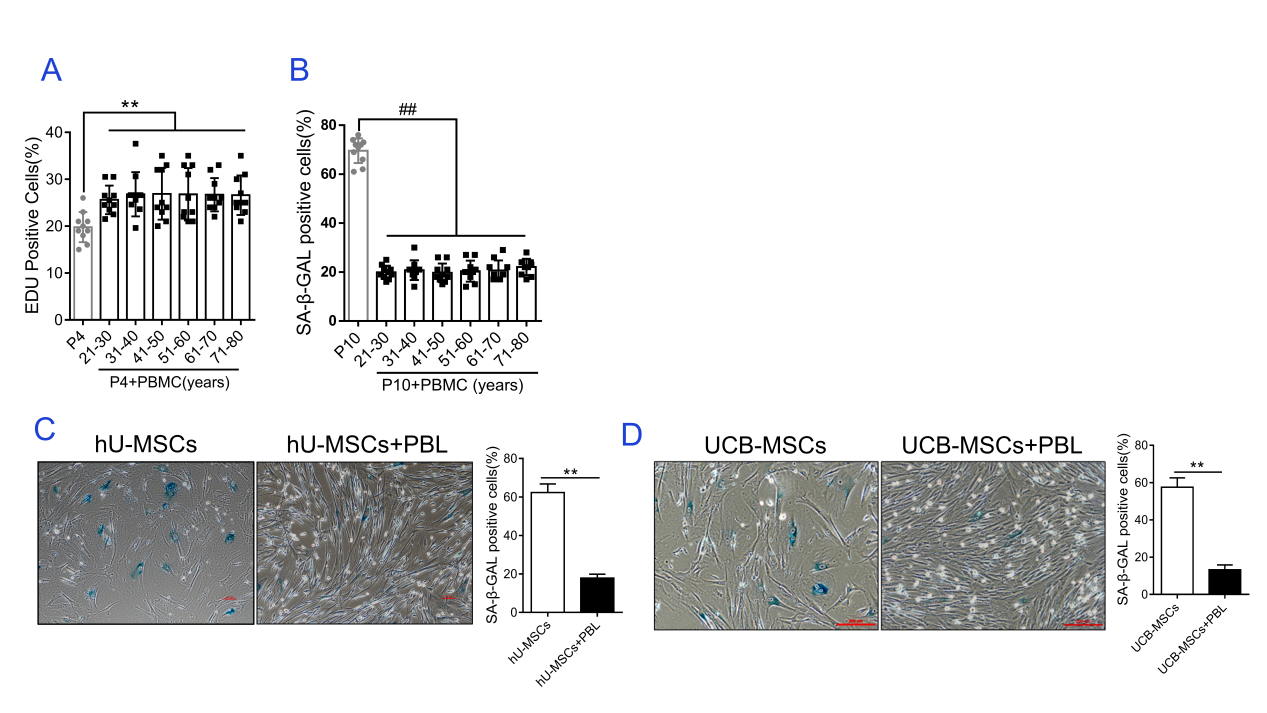


**Figure S1. The proproliferation and anti-senescence effects of PBMCs and PBLs were analyzed in the coculture system.** (A) PBMCs from donors of different ages promoted the proliferation of P4 hAMSCs with similar efficacy. (B) PBMCs from donors of different ages reversed the senescence phenotype of P10 hAMSCs with similar efficacy. The percentage of SA-β-gal-positive cells in the P10 group was 69.75±4.79%. (C) Analysis of SA-β-gal staining in P10 hU-MSCs. P10 hU-MSCs were co-cultured with PBLs for 3 days. (D) Analysis of SA-β-gal staining in P10 UCB-MSCs. P10 UCB-MSCs were co-cultured with PBLs for 3 days. Note: In Fig.S1A-B, PBLs for each age cohort were collected from ten distinct peripheral blood donors; hAMSCs were isolated from three independent amniotic membrane donors. hU-MSCs: human umbilical cord mesenchymal stem cells; UCB-MSCs: human umbilical cord blood mesenchymal stem cells. Statistical significance compared with the P4 group is denoted as ^**^*P* < 0.01; that compared with the P10 group is denoted as ^##^*P* < 0.01.


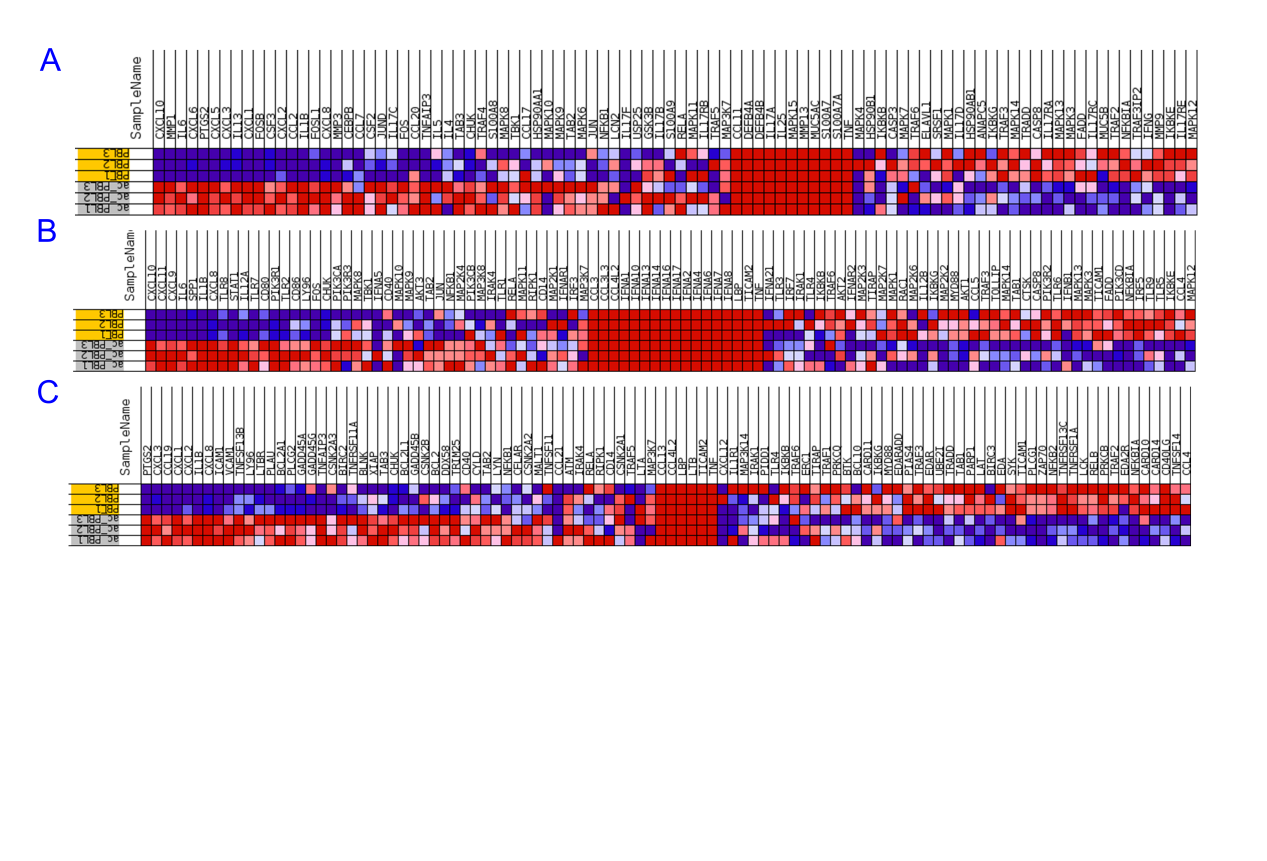


**Figure S2. Coculture with hAMSCs activated the secretion profile of PBLs.** (A) Clustering of differentially expressed genes in the IL-17 signaling pathway. (B) Clustering of differentially expressed genes in the Toll-like receptor signaling pathway. (C) Clustering of differentially expressed genes in the NF-kappa B signaling pathway.


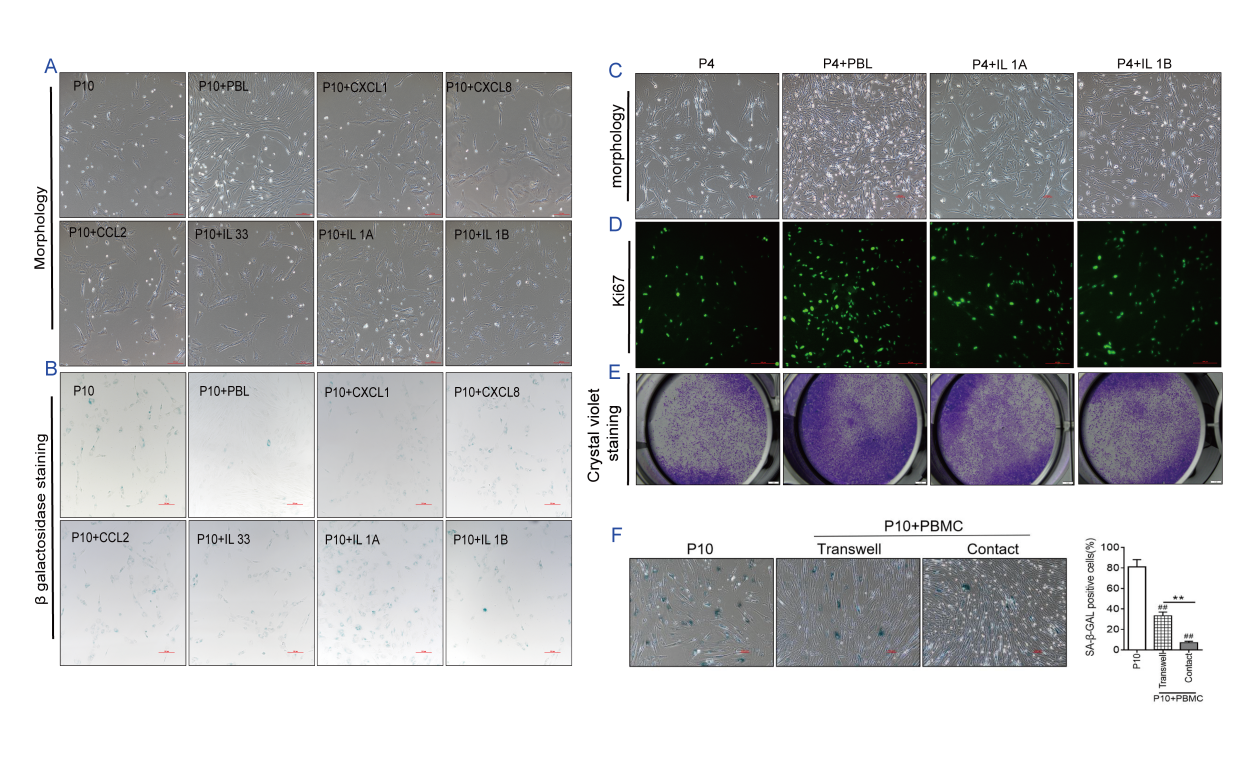


**Figure S3. Effects of cytokines on hAMSC phenotype and characteristics.** (A) Effects of cytokines on the morphology of P10 hAMSCs. (B) Analysis of SA-β-gal staining in P10 hAMSCs. Scale bar: 100 µm. (C) Effects of cytokines on the morphology of P4 hAMSCs. (D) Ki67 Expression in P4 hAMSCs was analyzed via immunofluorescence staining. (E) Cell density was analyzed via crystal violet staining. (F) Screening of co-culture modes of PBMCs and P10 hAMSCs. Note: P4: hAMSCs were expanded in vitro up to the fourth generation; P4 + PBL: hAMSCs from the fourth passage were cocultured with PBLs at a ratio of 1:100. P10: hAMSCs were expanded in vitro up to the tenth generation; P10 + PBL: hAMSCs from the tenth passage were cocultured with PBL at a ratio of 1:100; SA-β-gal: β-galactosidase.

**
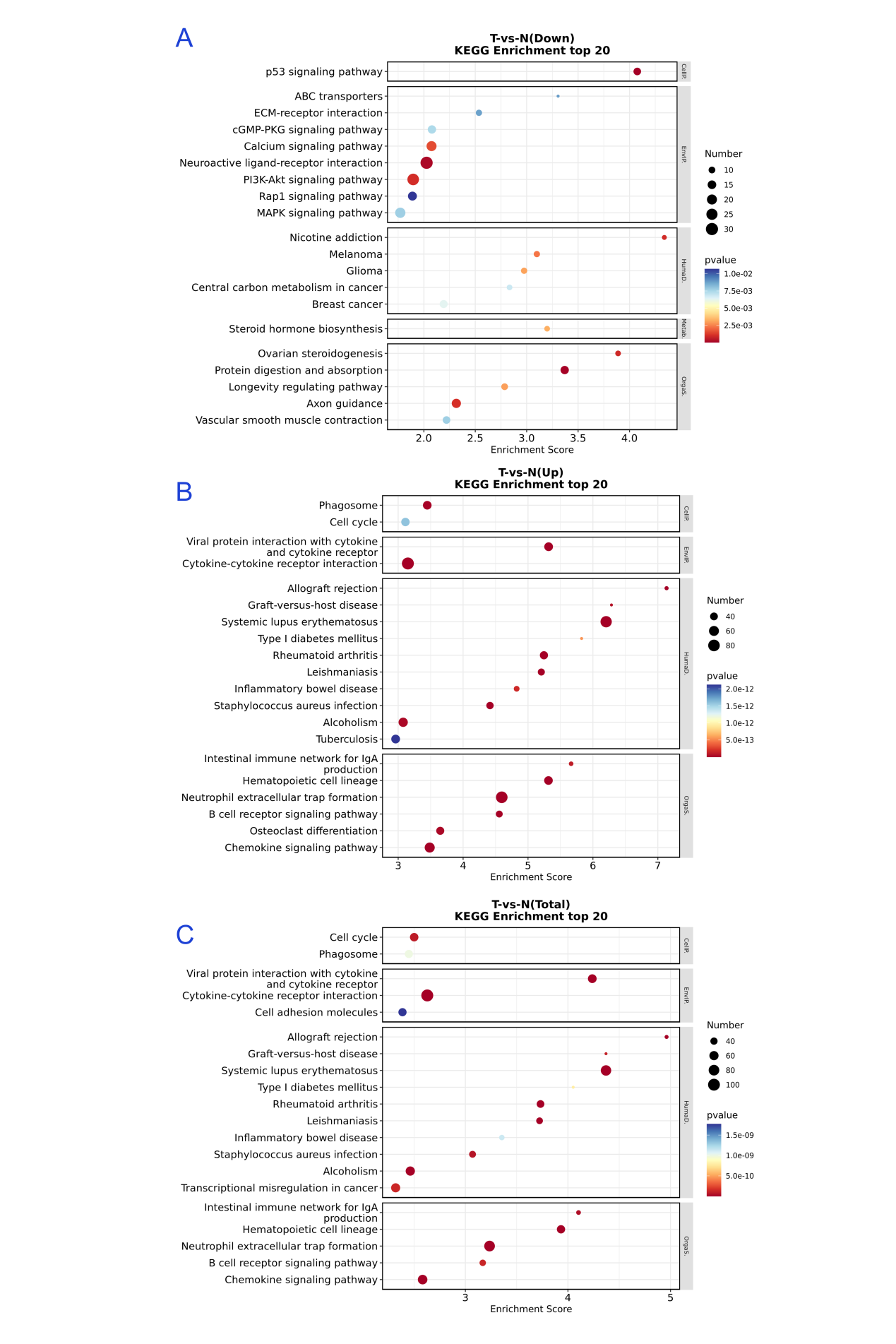
**

**Figure S4. KEGG enrichment analysis of the RNA-seq data.** (A) Clustering of differentially expressed genes in the IL-17 signaling pathway. (B) Clustering of differentially expressed genes in the Toll-like receptor signaling pathway. (C) Clustering of differentially expressed genes in the NF-kappa B signaling pathway.


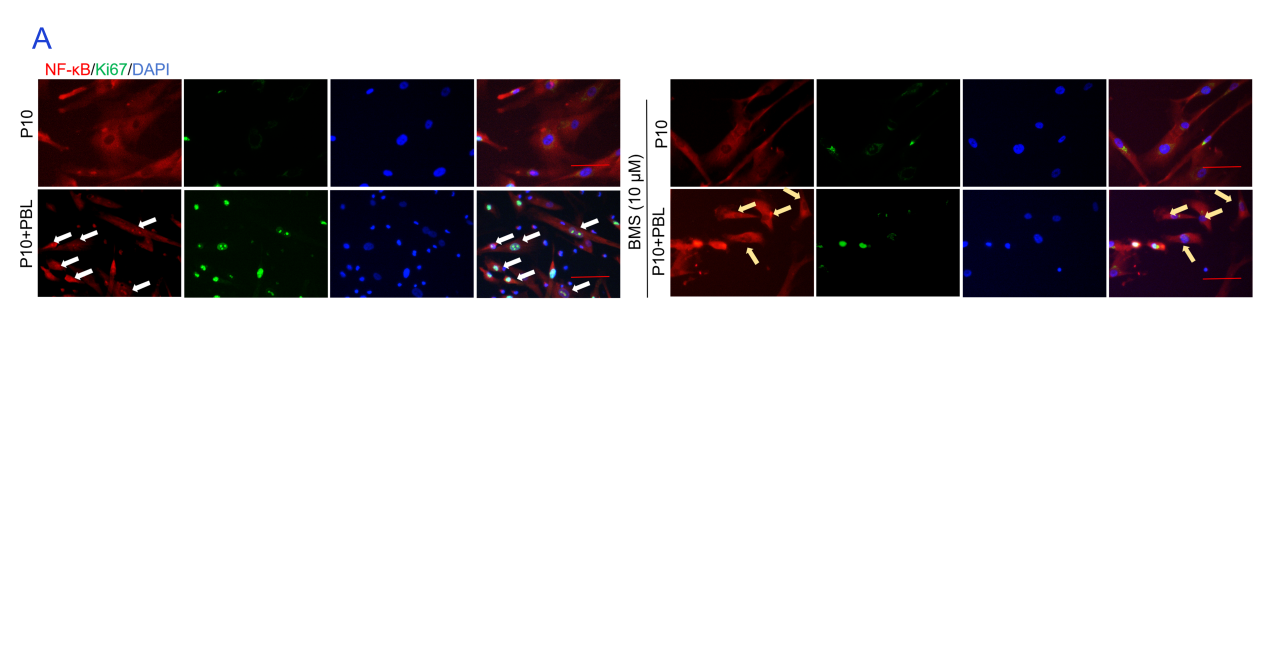


**Figure S5.** Expression of NF-κB in P10 hAMSCs. The white arrow represents NF-κB activation. The yellow arrow represents BMS-345541 inhibition of NF-κB activation. Scale bar: 100 μm.
