# Supplementary material for: Rejuvenation of mesenchymal stem cells by human peripheral blood lymphocytes
Source: BMC Biol. 2025 Nov 25;23:370. doi: 10.1186/s12915-025-02472-9 (PMC12750958; doi:10.1186/s12915-025-02472-9)
Supplement: Supplementary file 3 — Additional file 3: Table S1. Key resource information used in this study. [file 12915_2025_2472_MOESM3_ESM.docx]

**Supplementary Table**

**Table S1.** Key resource information used in this study.

| **REAGENT or RESOURCE** | **SOURCE** | **IDENTIFIER** |
| --- | --- | --- |
| **Antibodies** | | |
| Rabbit anti-Vimentin | Abcam | Cat# ab16700; RRID:AB_443435 |
| Rabbit anti-Cytokeratin 19 | Abcam | Cat# ab76539; RRID:AB_1523469 |
| Rabbit anti-Occludin | Abcam | Cat# ab216327; RRID:AB_2737295 |
| Rabbit anti-p21 | Abcam | Cat# ab109520; RRID:AB_10860537 |
| Rabbit anti-Anti-p16INK4a | Abcam | Cat# ab270058 |
| Rabbit anti-Fas | Abcam | Cat# ab133619; RRID:AB_2940837 |
| Mouse anti-Fas Ligand | Abcam | Cat# ab303666 |
| Rabbit anti-Sox2 | Abcam | Cat# ab92494; RRID:AB_10585428 |
| Rabbit anti-Nanog | Abcam | Cat# ab109250; RRID:AB_10863442 |
| Mouse anti-Caspase-8 | Cell signaling technology | Cat# 9746; RRID:AB_2275120 |
| Mouse anti-NF-κB p65 | Cell signaling technology | Cat# 6956; RRID:AB_10828935 |
| Mouse anti-Claudin-2 | Santa Cruz Biotechnology | Cat# Sc-293233 |
| Rabbit anti-Granzyme B | Proteintech | Cat# 13588-1-AP; RRID:AB_2114429 |
| Rabbit anti-Perforin | Proteintech | Cat# 14580-1-AP; RRID:AB_10639524 |
| Mouse anti-Beta actin | Proteintech | Cat# 66009-1-Ig; RRID:AB_2687938 |
| Mouse anti-GAPDH | Proteintech | Cat# 60004-1-Ig; RRID:AB_2107436 |
| Rabbit anti-Histone-H3 | Proteintech | Cat# 17168-1-AP; RRID:AB_2716755 |
| Mouse anti-Claudin-1 | Huabio | Cat# RT1141 |
| Rabbit anti-Caspase-3 | Huabio | Cat# ET1602-39; RRID:AB_3069652 |
| Rabbit anti-IKB alpha | Huabio | Cat# ET1603-6; RRID:AB_3065034 |
| Rabbit anti-Phospho-IKB alpha (S36) | Huabio | Cat# HA721802; RRID:AB_3072914 |
| FITC anti-human HLA-DR, DP, DQ | Biolegend | Cat# 361705; RRID:AB_2563191 |
| PerCP/Cyanine 5.5 anti-human CD8 | Biolegend | Cat# 260230; RRID:AB_2941450 |
| **Chemicals, peptides, and recombinant proteins** | | |
| Penicillic acid (PCA) | MCE | HY-N6777 |
| BMS-345541 | Sigma | B9935 |
| Dextran sulfate sodium salt | Mpbiochina | 9011-18-1 |
| **Critical commercial assays** | | |
| Human MSC analysis kit | BD biosciences | 562245 |
| senescence-associated β-galactosidase staining kit | Beyotime | C0602 |
| Human Telomere Length Quantification qPCR Assay kit | ELK biotechnology | EQ022 |
| EdU apollo488 in vitro kit | Ribobio | C10310-3 |
| DNA content detection kit | Solarbio | CA1510 |
| ROS Assay kit | Solarbio | CA1410 |
| CellEvent™ Senescence Green Flow Cytometry Assay kit | Invitrogen | C10841 |
| CFDA, SE Cell Proliferation and Tracer Assay kit | Solarbio | CA1200 |
| **Experimental models: Cell lines** | | |
| Human amniotic mesenchymal stem cells | Yuan et al. ^[22]^ | N/A |
| Human peripheral blood mononuclear cell | Renner et al. ^[29]^ | N/A |
| Human peripheral blood monocytes | Dagur et al. ^[30]^ | N/A |
| Human peripheral blood lymphocytes | Dagur et al. ^[30]^ | N/A |
| A549 cells | Derived in house | N/A |
| **Experimental models: Organisms/strains** | | |
| C57BL/6J | Changsha Tianqin Biotechnology Co., Ltd | SCXK 2019-0014 |
| Nude mice | Spiff Beijing Biotechnology | SCXK 2019-0010 |
| **Software and algorithms** | | |
| Adobe Prime | www.adobe.com | N/A |
