# Supplementary material for: Rejuvenation of mesenchymal stem cells by human peripheral blood lymphocytes
Source: BMC Biol. 2025 Nov 25;23:370. doi: 10.1186/s12915-025-02472-9 (PMC12750958; doi:10.1186/s12915-025-02472-9)

**Original uncropped Western blot images**

**Figure 1G**

β-actin


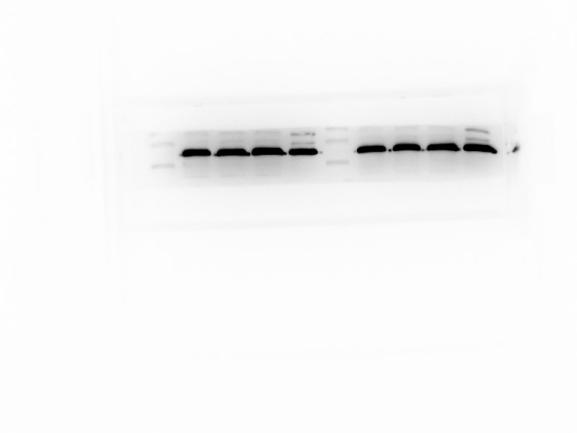

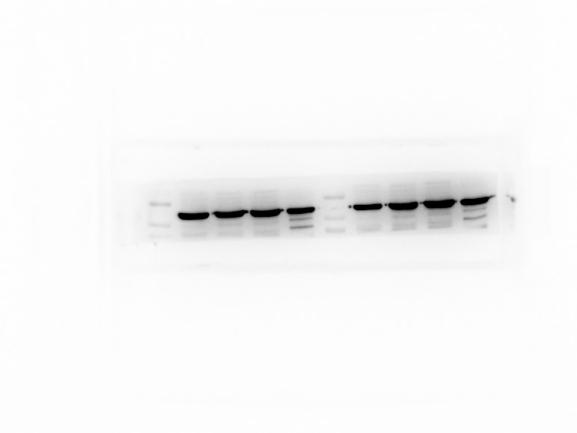


p16


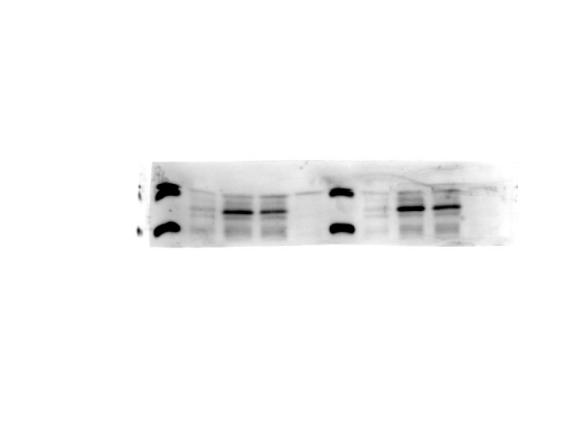

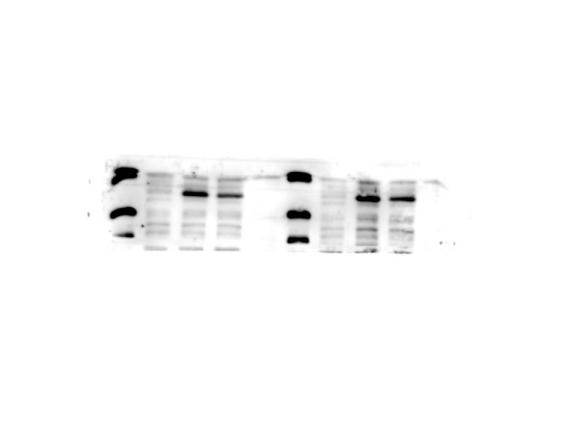


p21


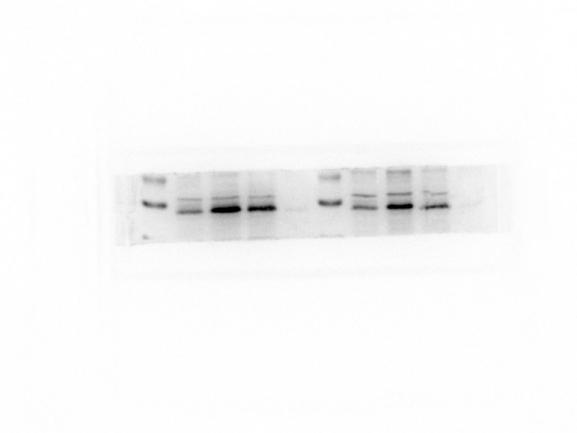

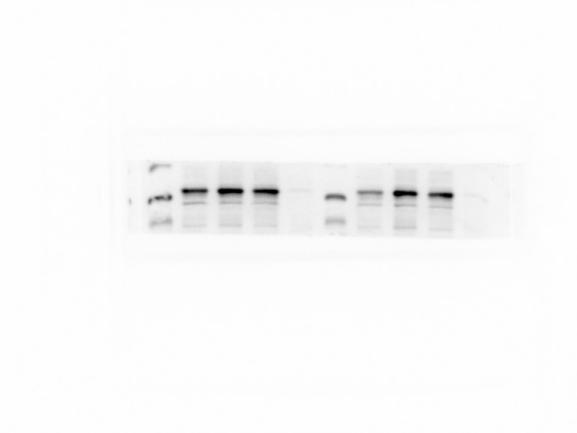


**Figure 1L**

β-actin-1/SOX2-1


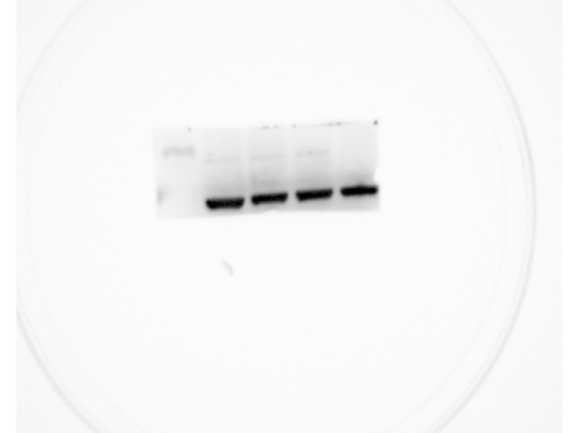

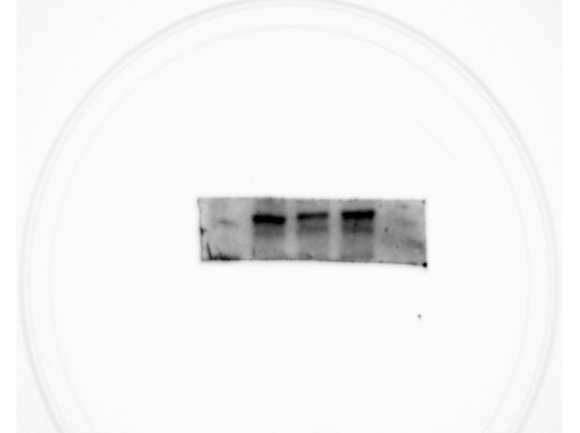


β-actin-2/SOX2-2


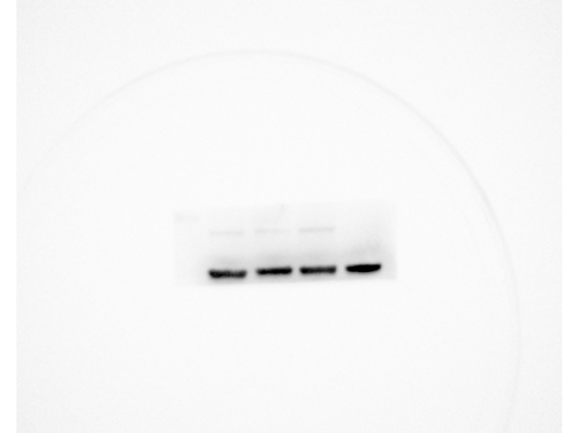

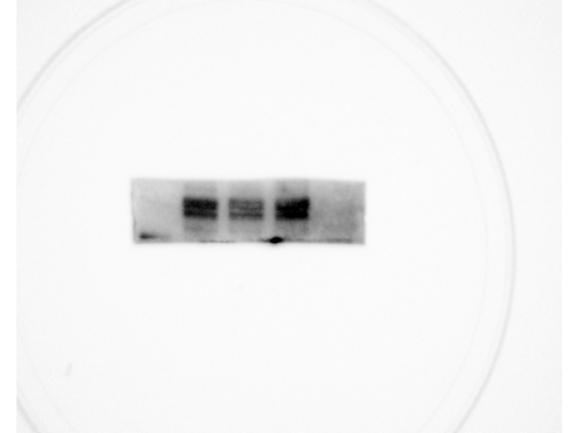


β-actin-3/SOX2-3


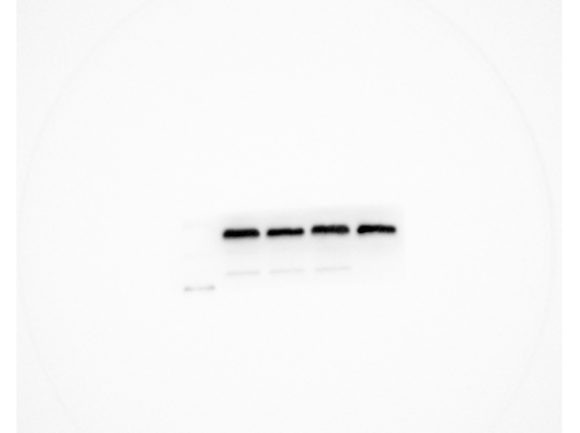

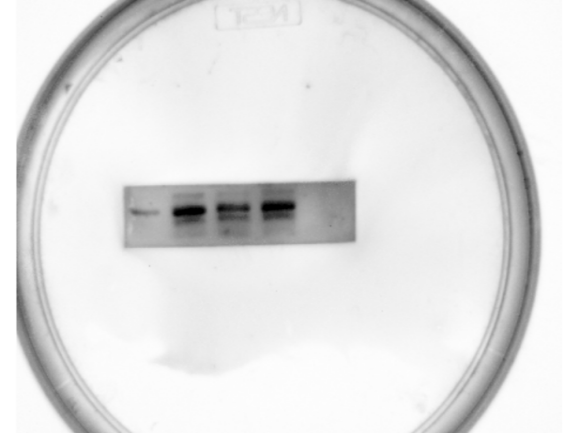


β-actin-1/Nanog-1


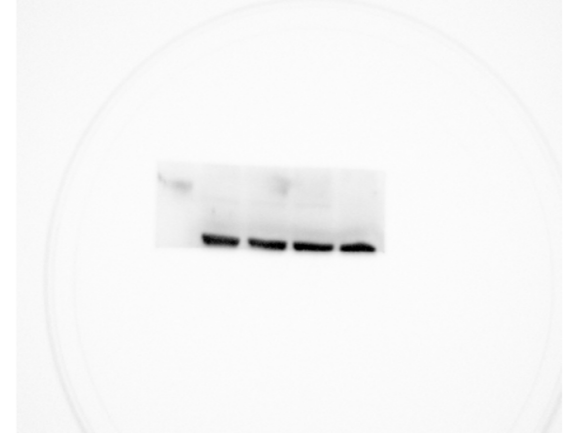

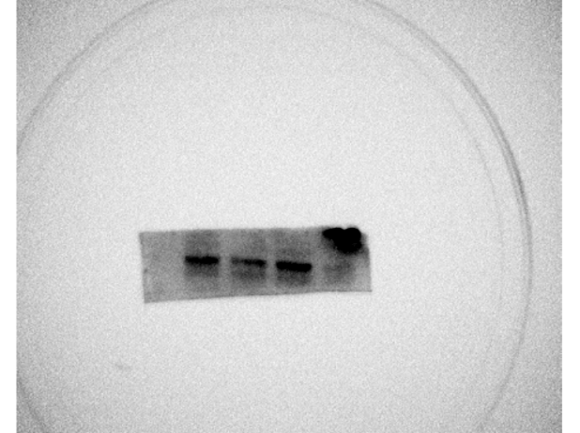


β-actin-2/Nanog-2


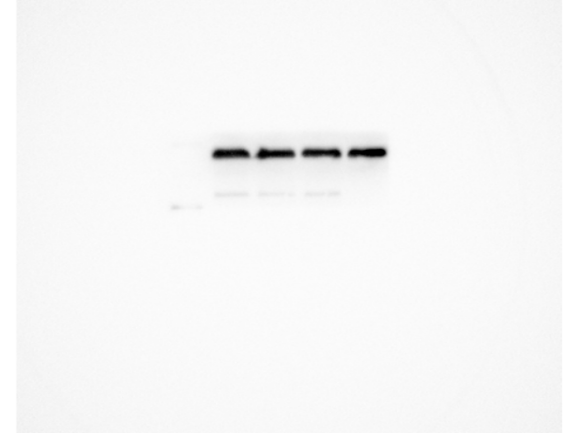

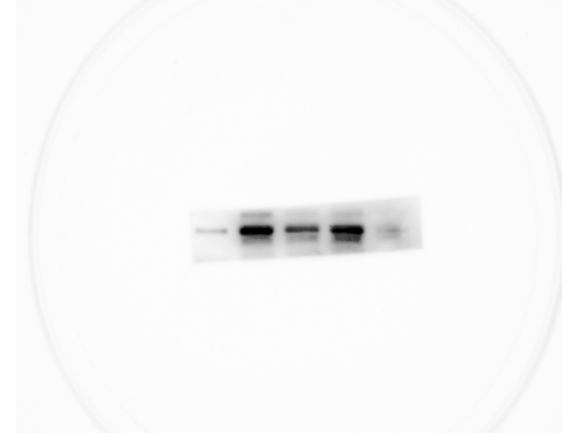


β-actin-3/Nanog-3


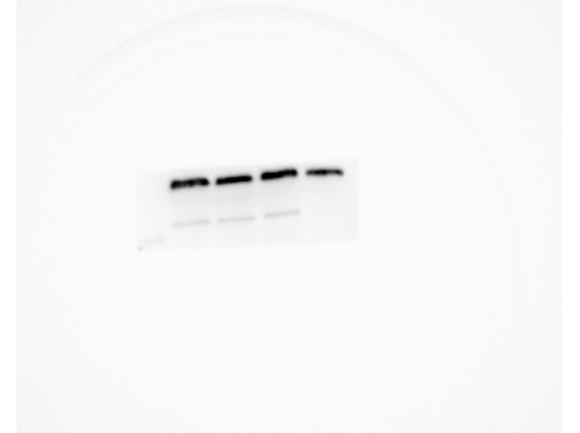

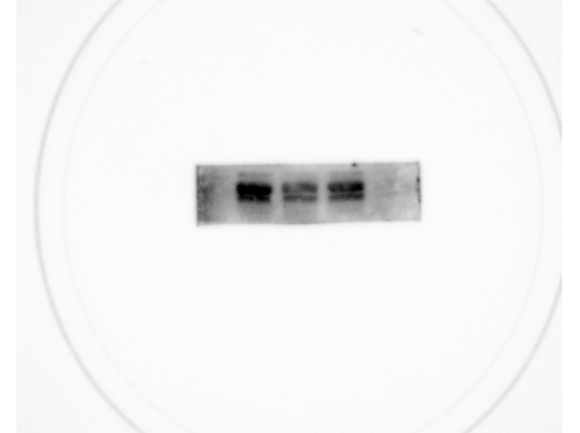


**Figure 4C**

β-actin/bax


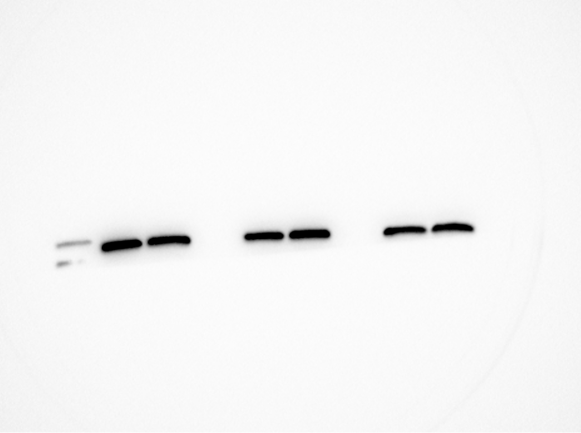

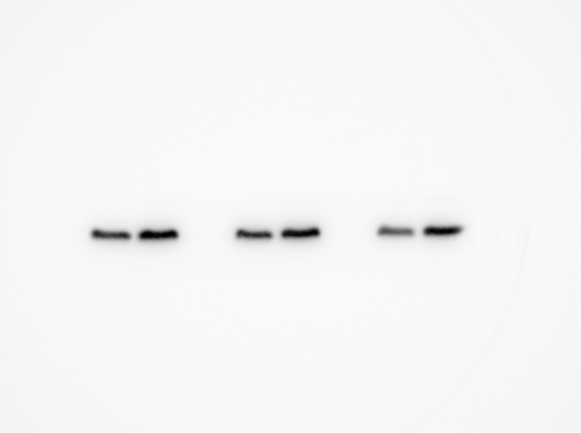


β-actin/bcl-2


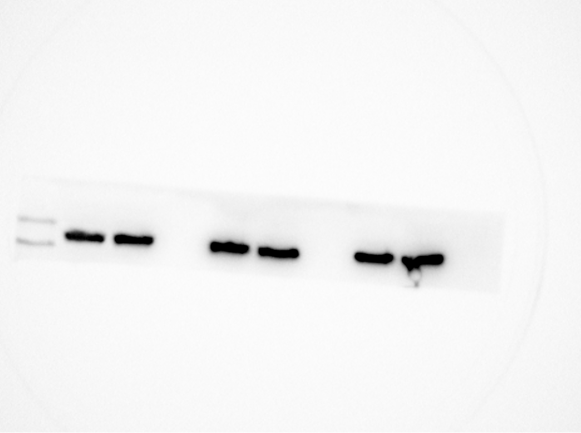

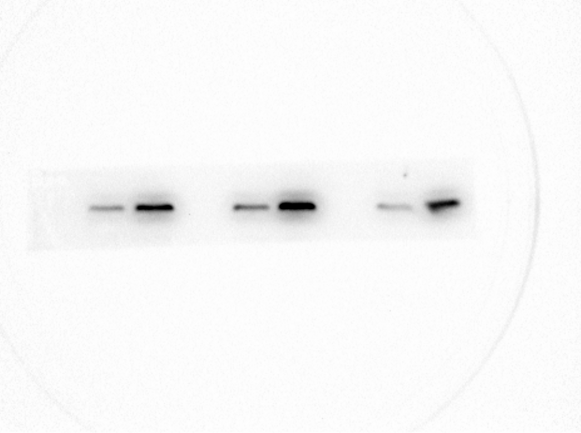


**Figure 4F**

β-actin/cyto c


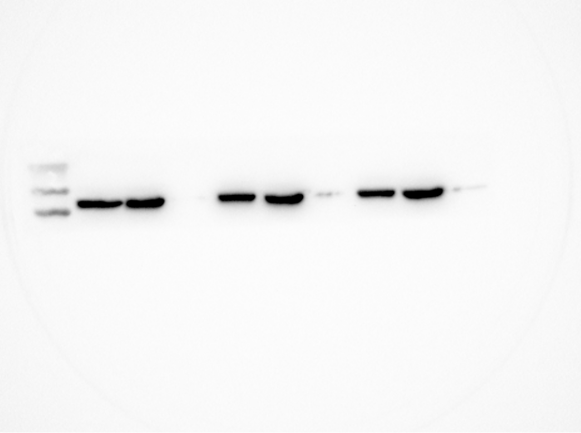

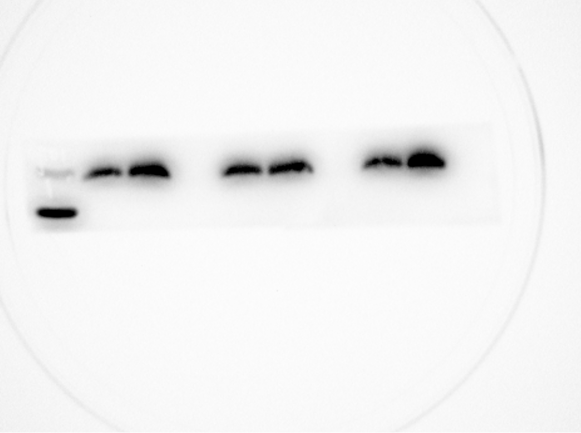


β-actin/SUMO1


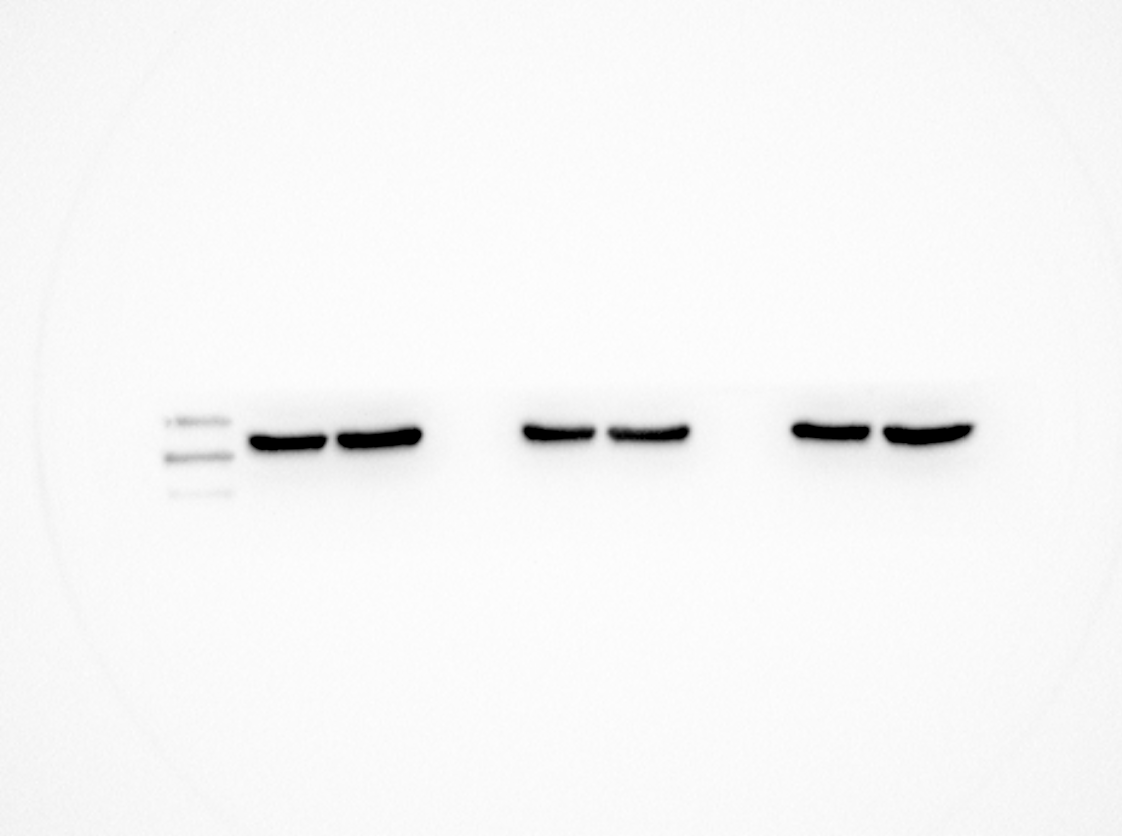

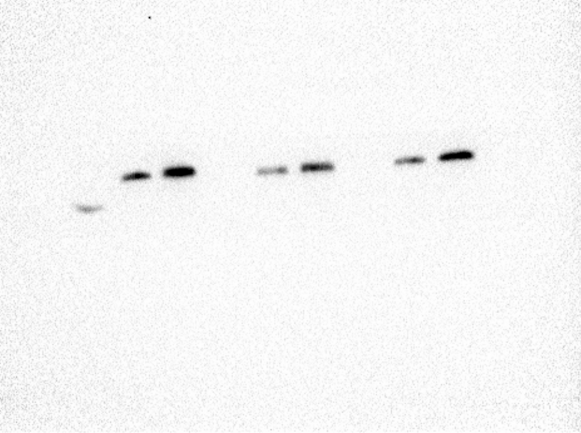


**Figure 4G**

Ip53/Ip-SUMO1


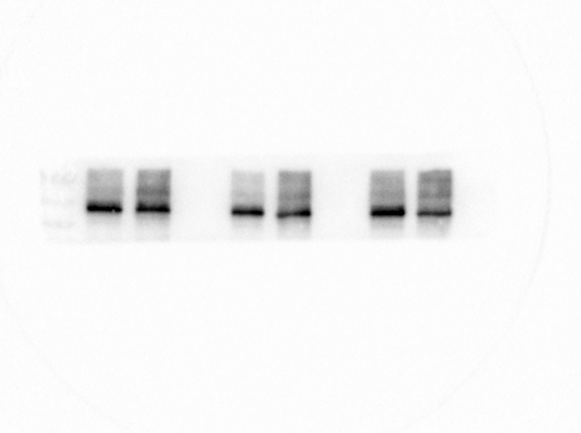

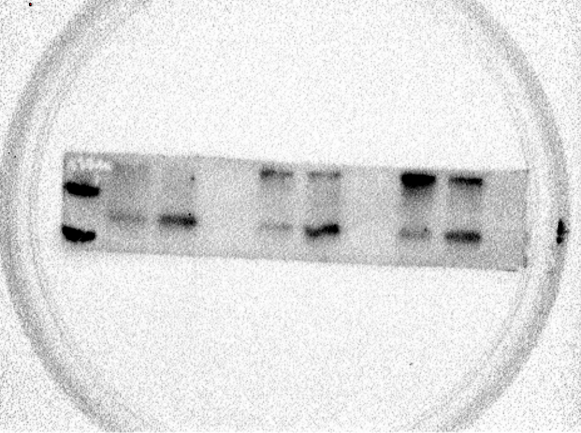


Input p53/sumo-1


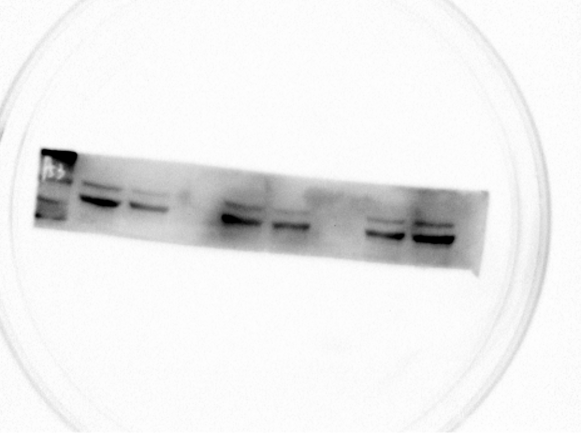

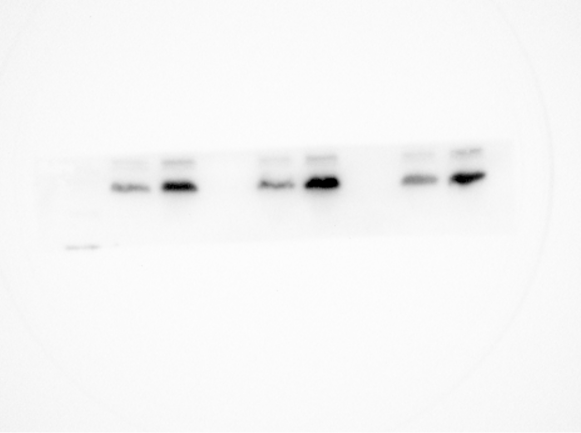


**Figure 4H**

β-actin/PIAS-1


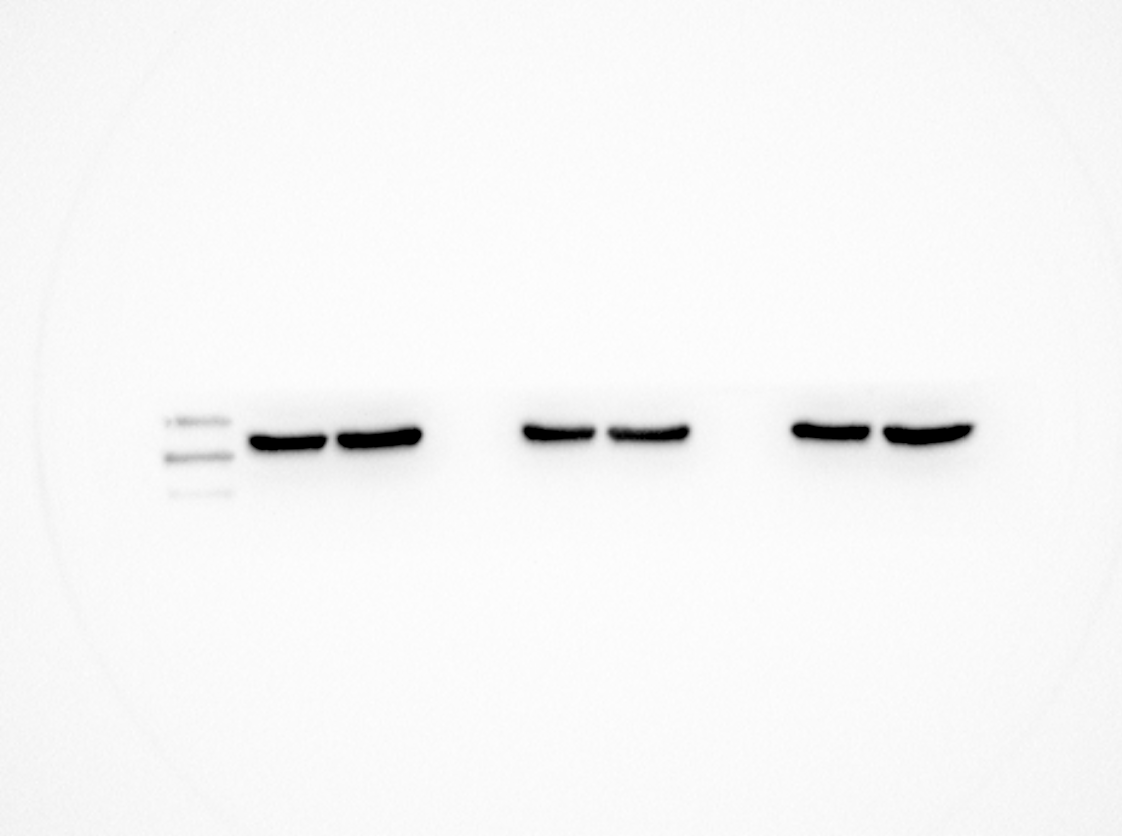

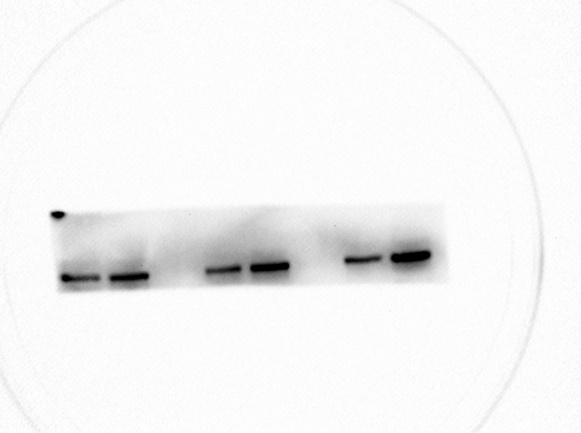


β-actin/SENP1


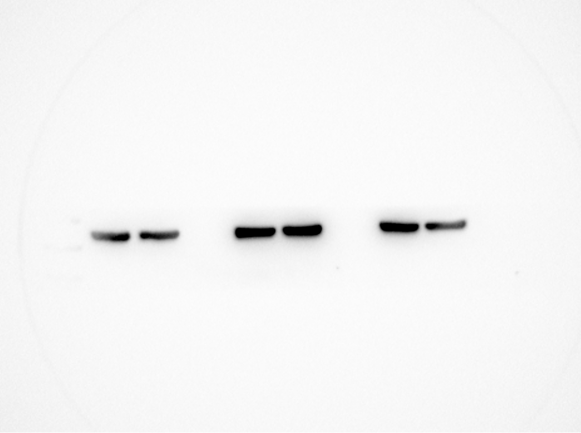

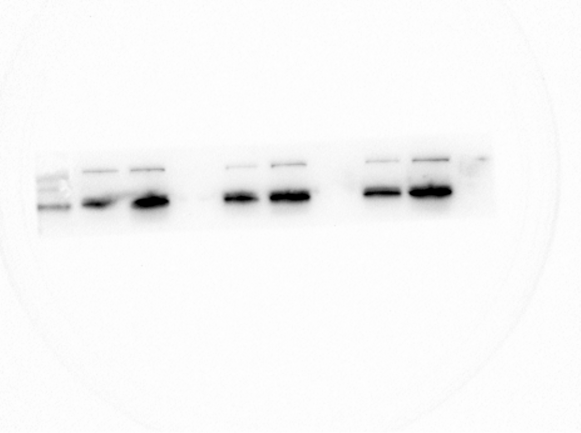


**Figure 5C**

GAPDH-1/Serpinb2-1


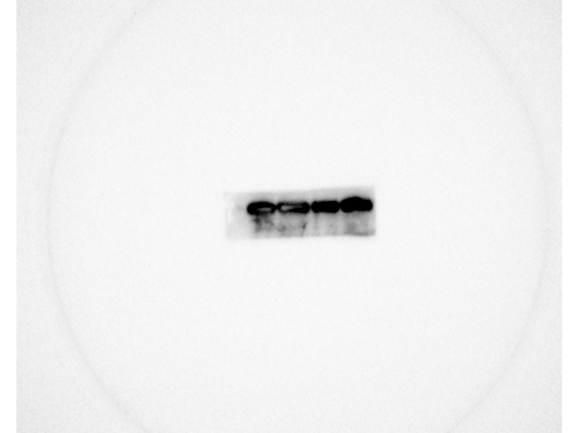

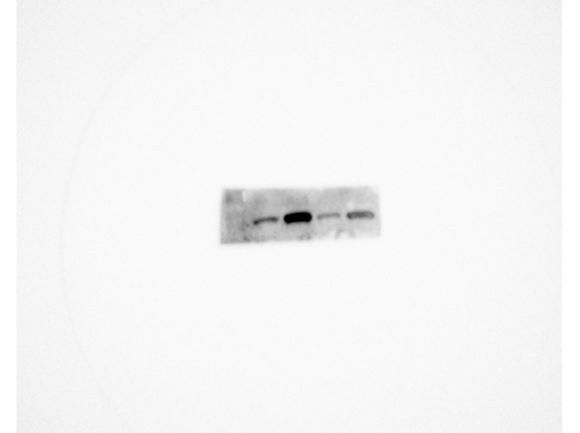


GAPDH-2/Serpinb2-2


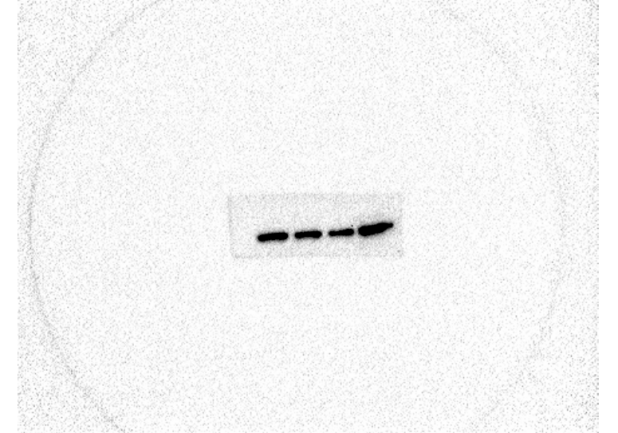

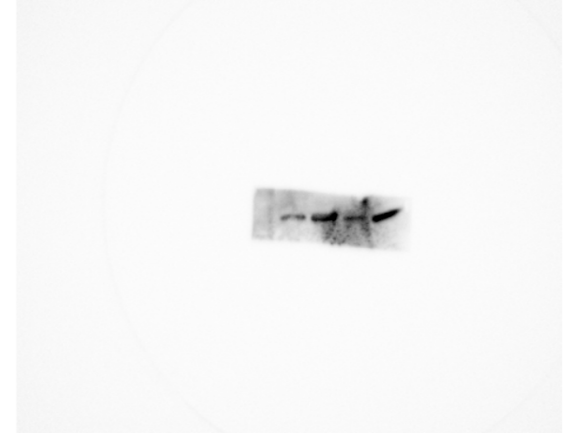


GAPDH-3/Serpinb2-3


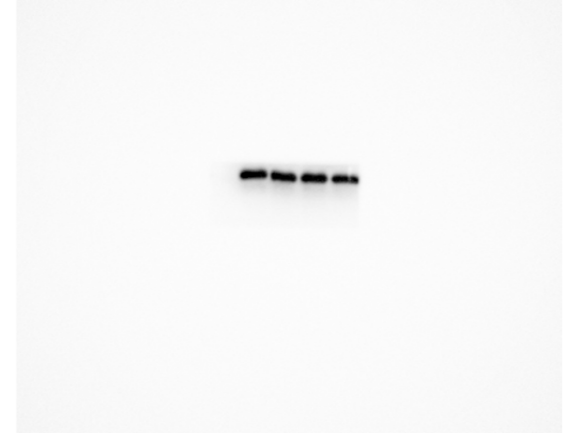

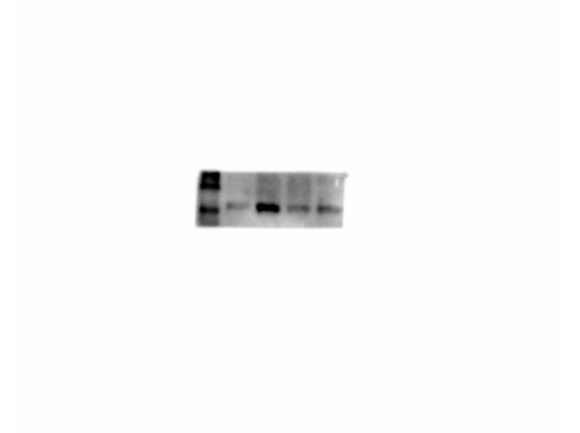


**Figure 5D**

β-actin-1/p21-1


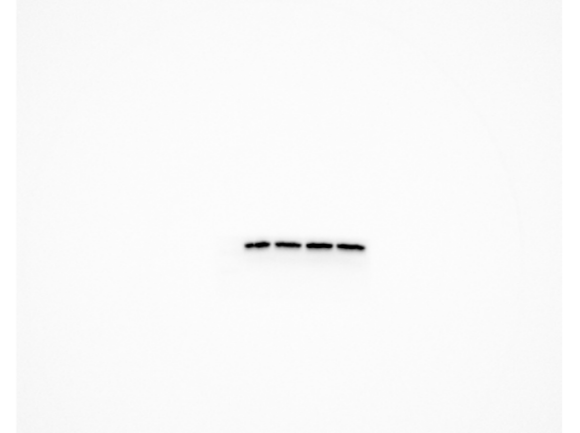

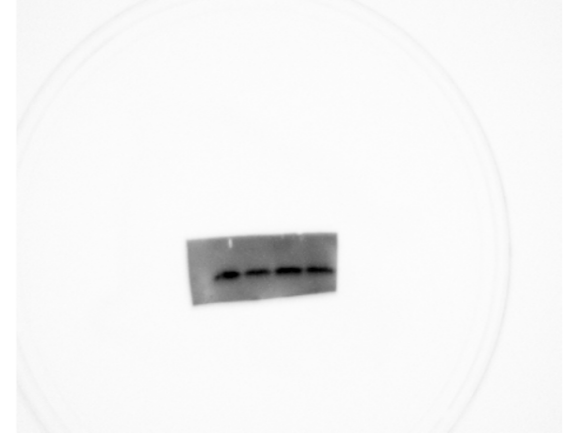


β-actin-2/p21-2


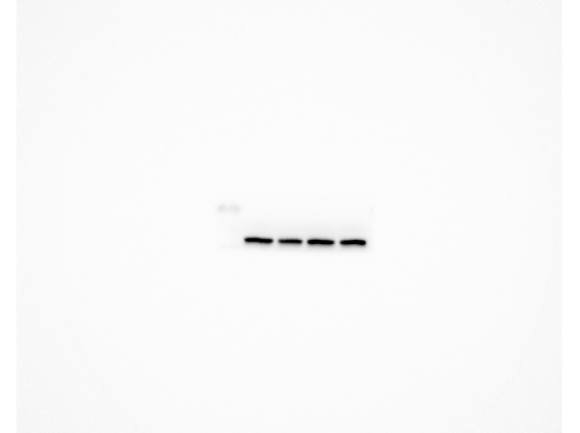

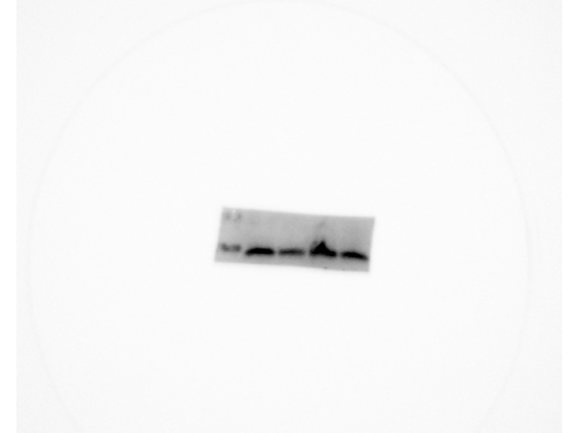


β-actin-3/p21-3


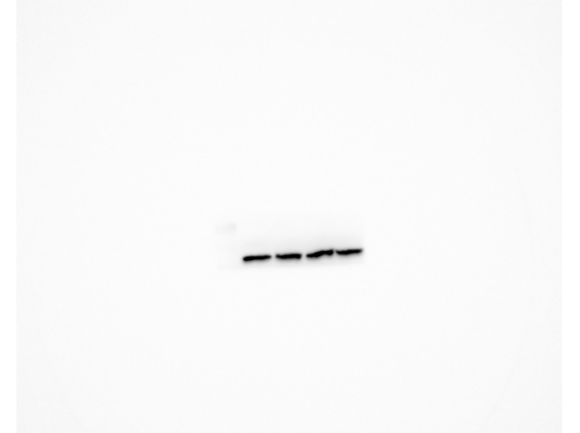

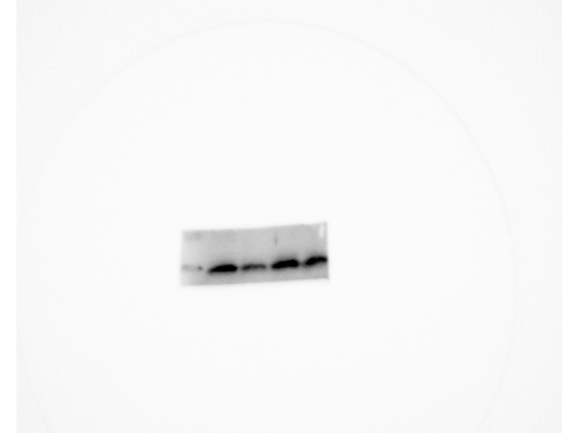


**Figure 5F**

β-actin-1/Nanog-1


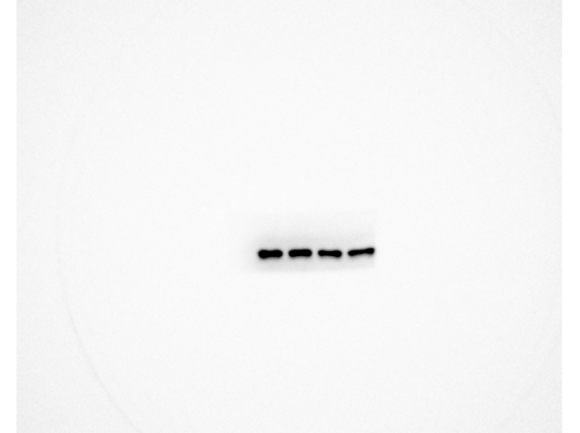

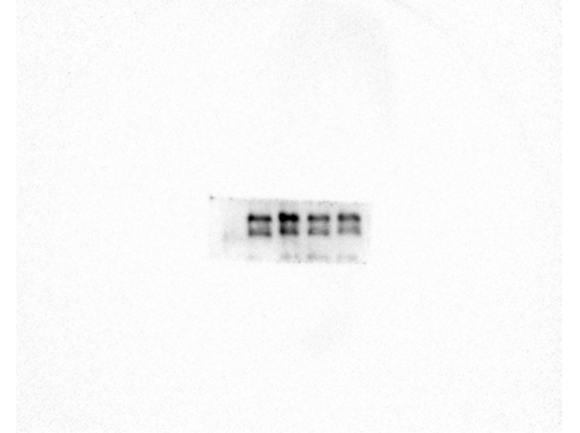


β-actin-1/Nanog-1


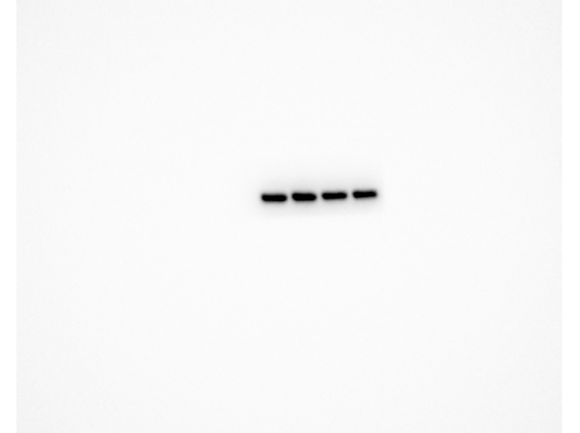

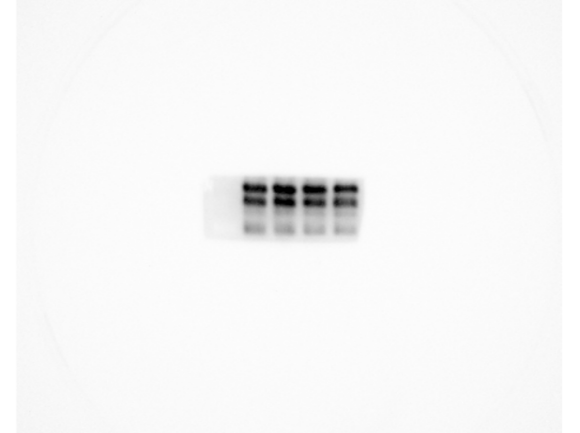


β-actin-1/Nanog-1


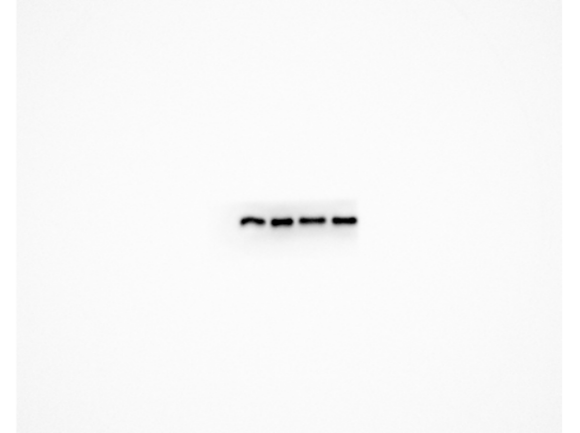

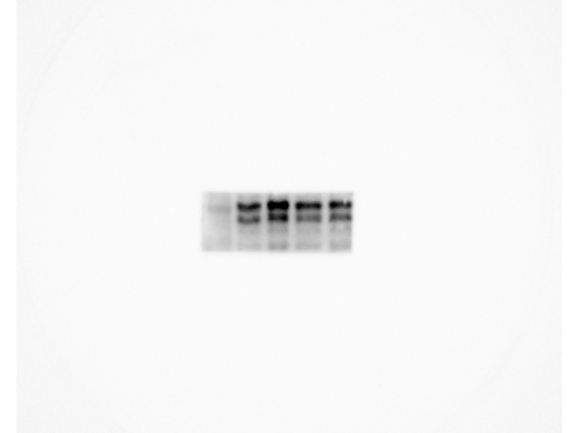


β-actin-1/Sox2-1


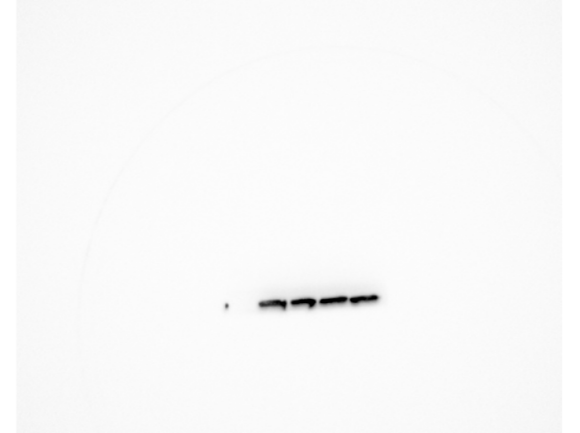

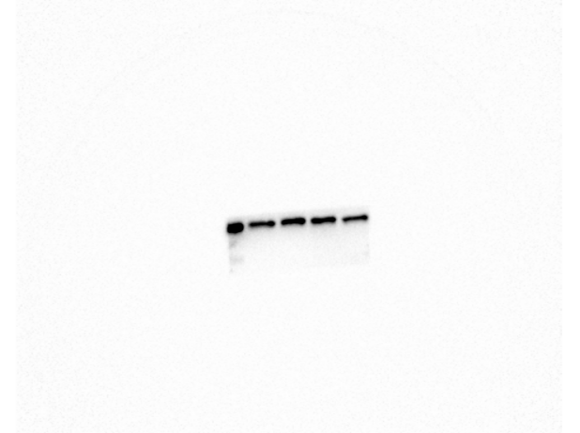


β-actin-2/Sox2-2


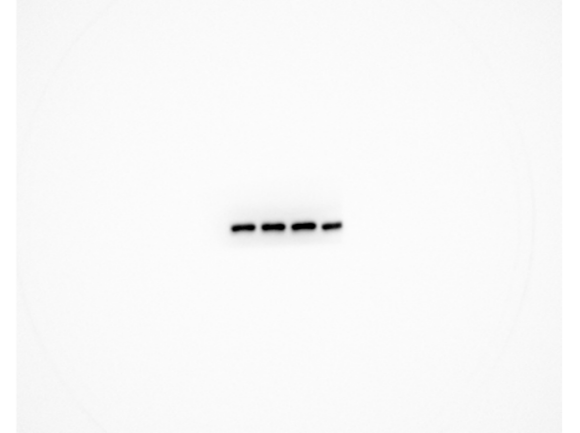

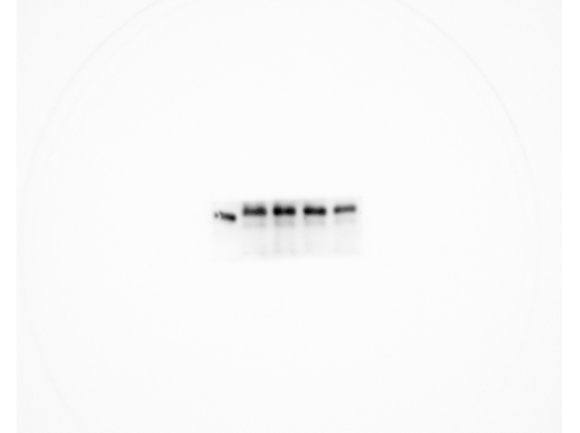


β-actin-3/Sox2-3


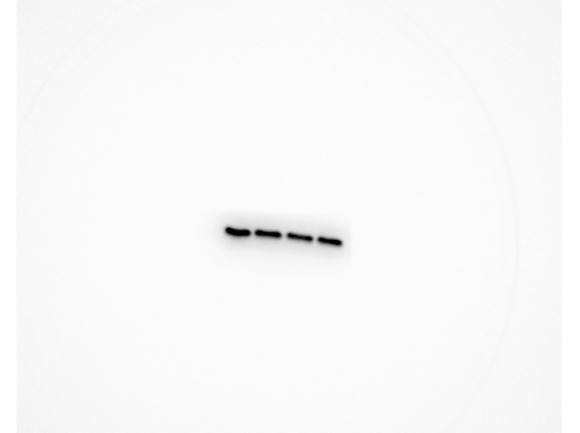

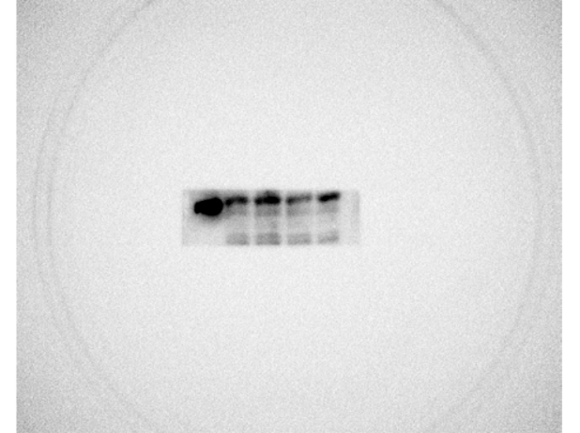


**Figure 5H**

β-actin-1/IκBα-1


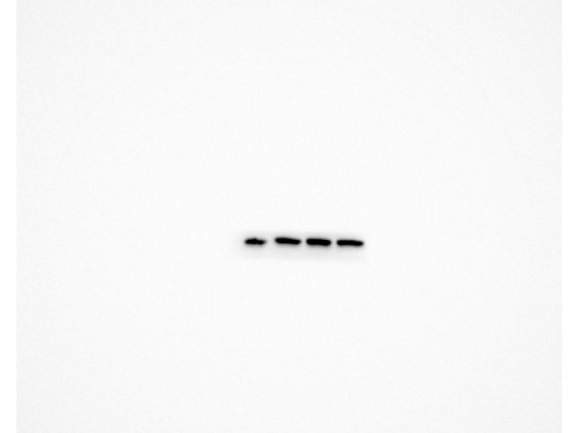

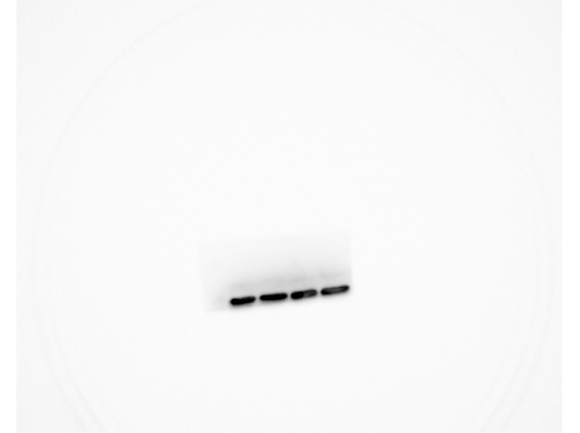


β-actin-2/IκBα-2


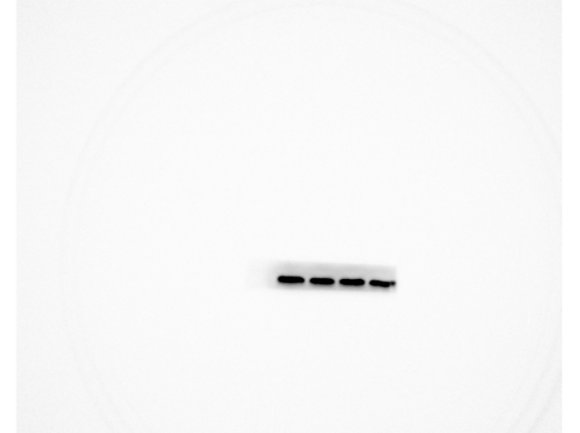

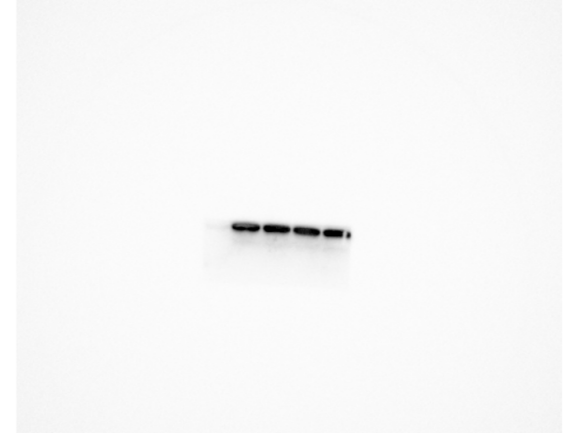


β-actin-3/IκBα-3


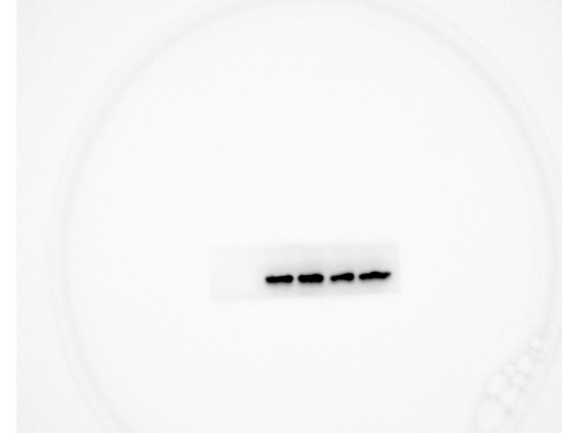

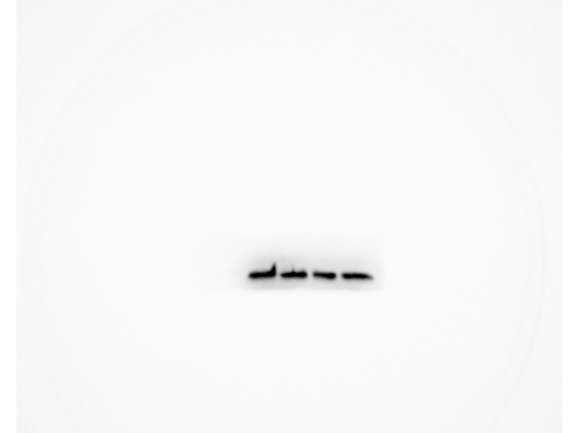


β-actin-1/p-IκBα-1


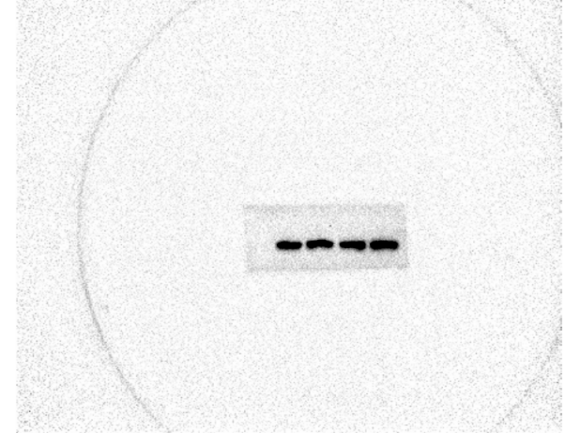

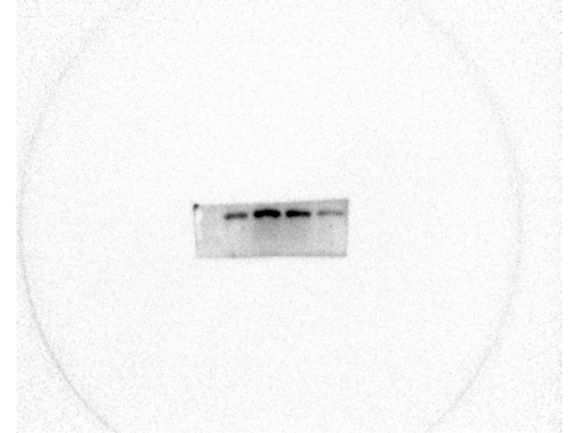


β-actin-2/p-IκBα-2


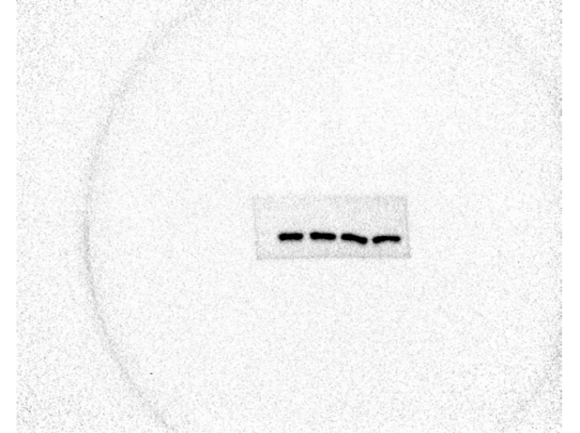

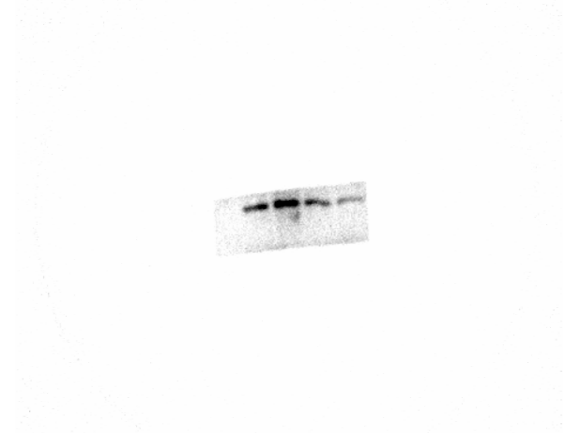


β-actin-3/p-IκBα-3


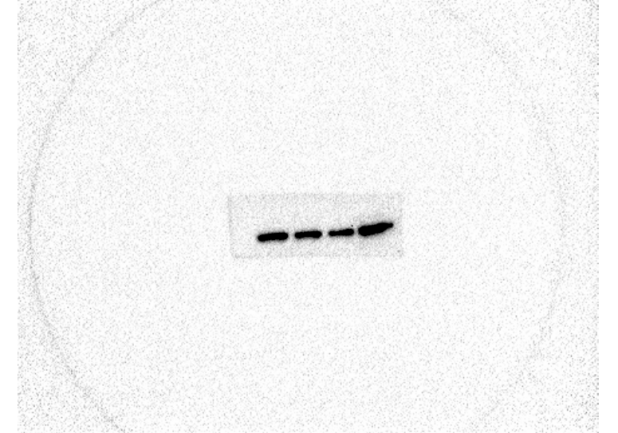

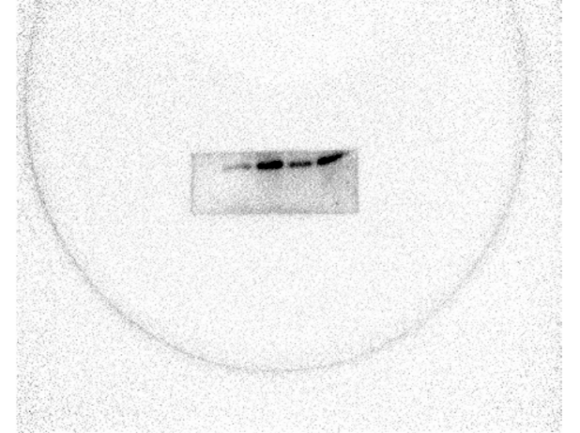


**Figure 6I**

β actin


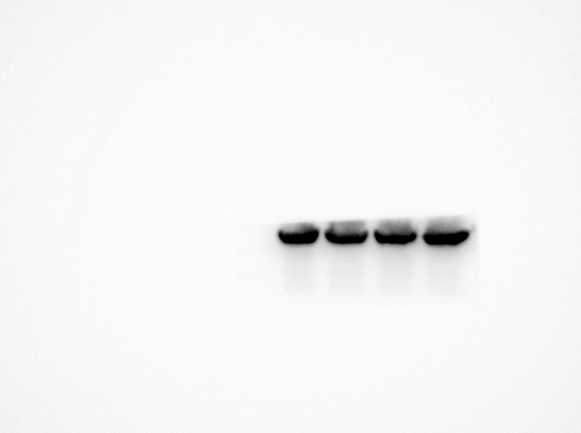

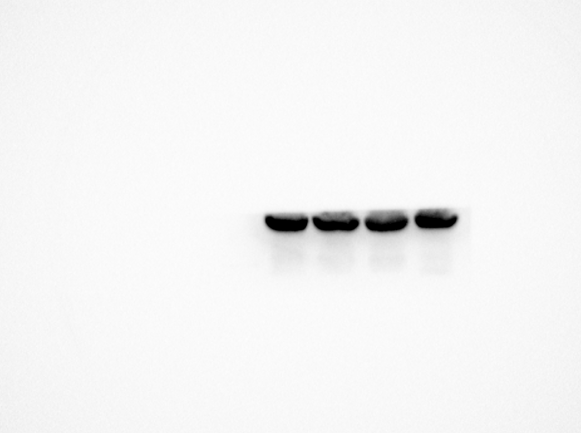


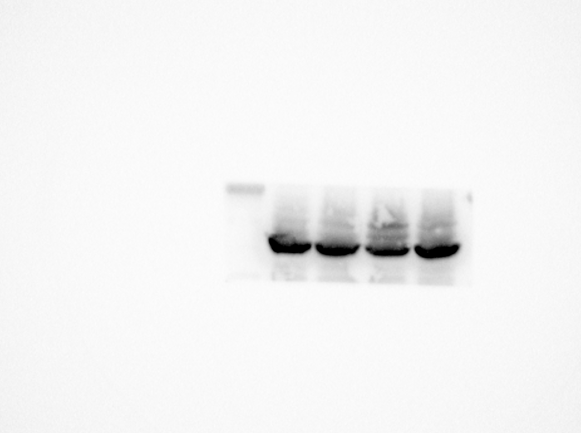

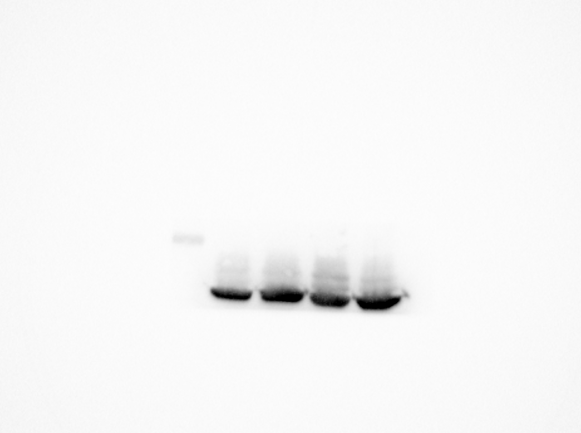


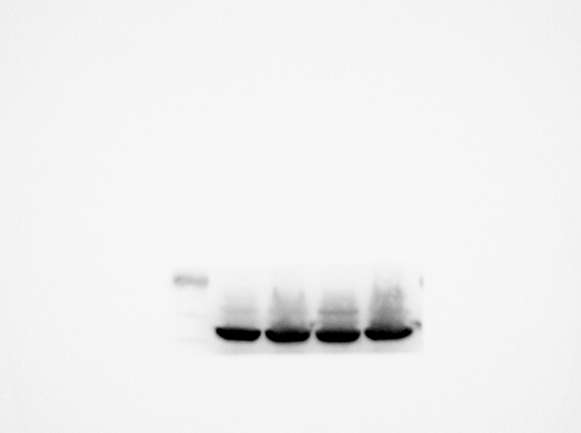

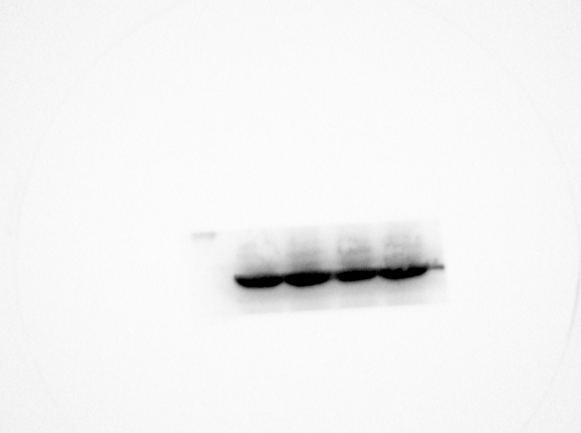


Occludin


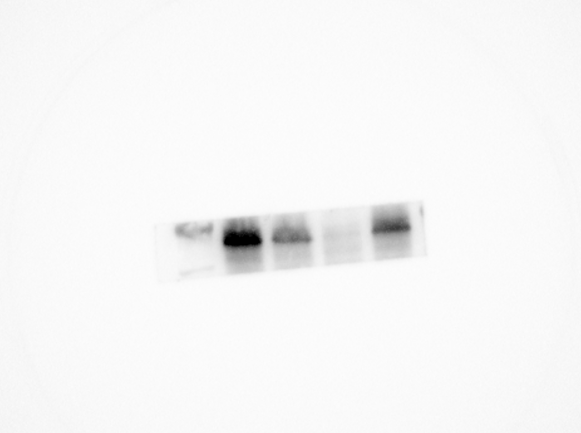

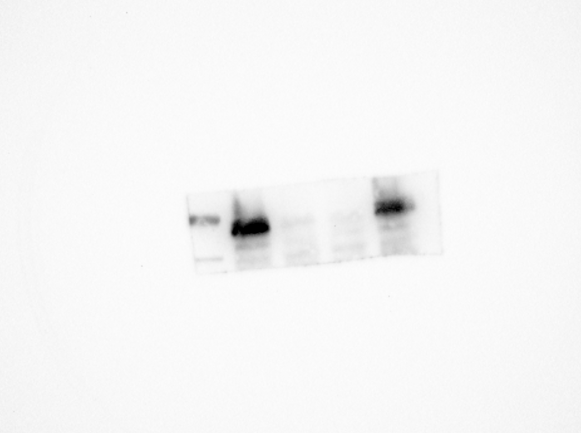


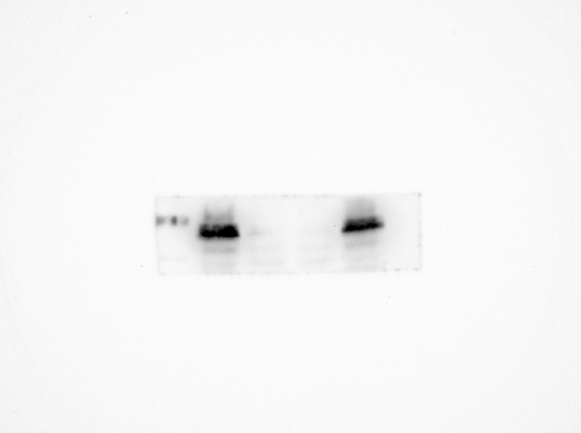

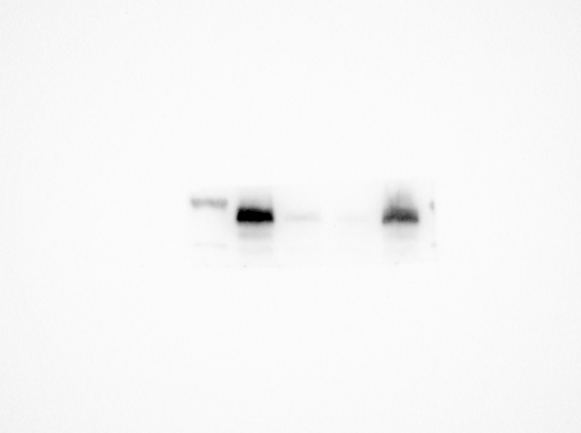


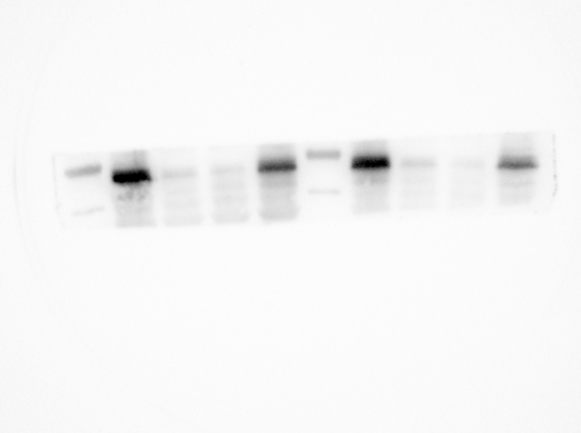

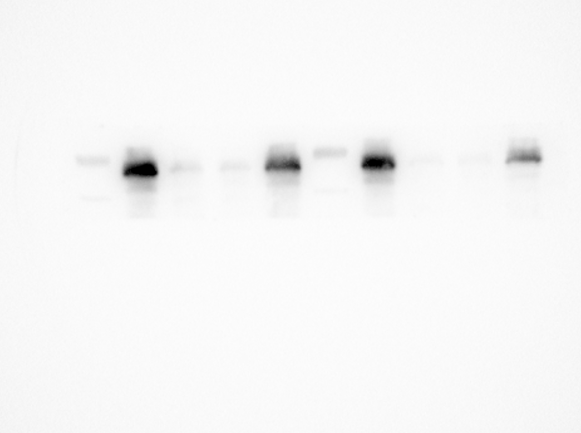


Caludin-1


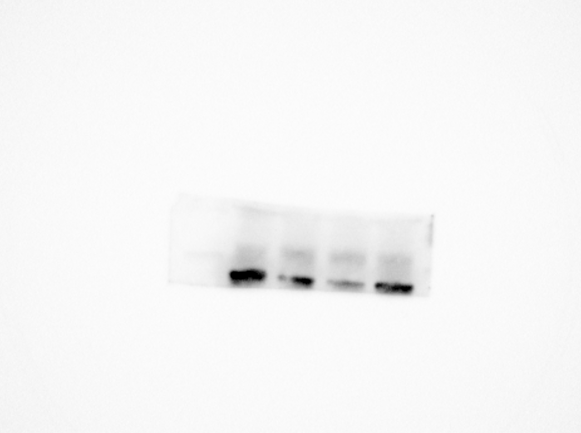

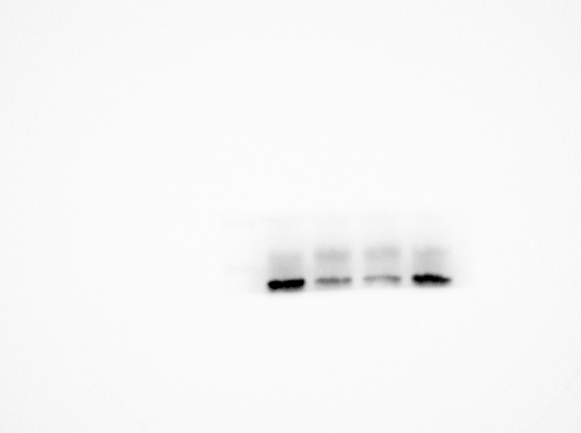


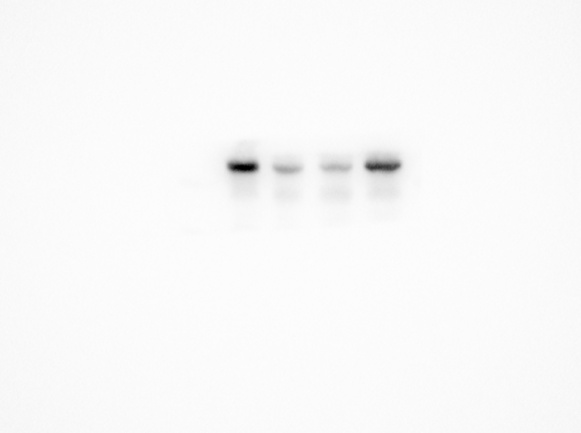

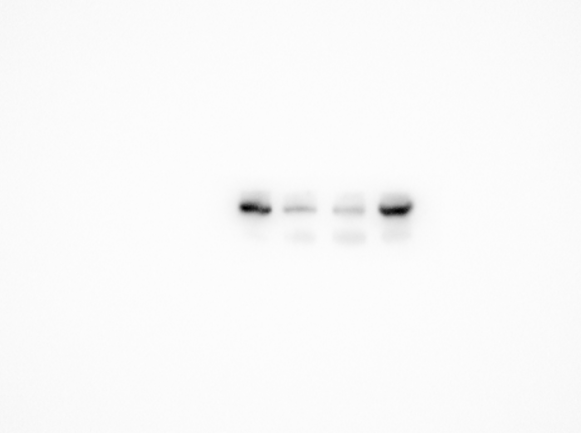


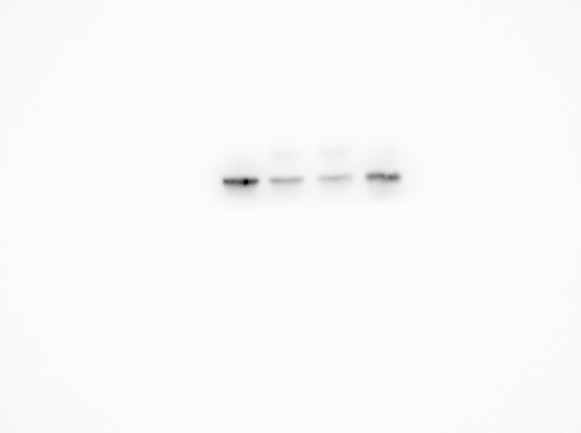

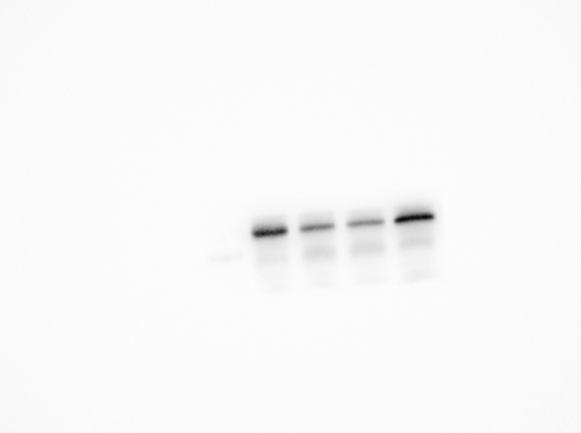


Claudin-2


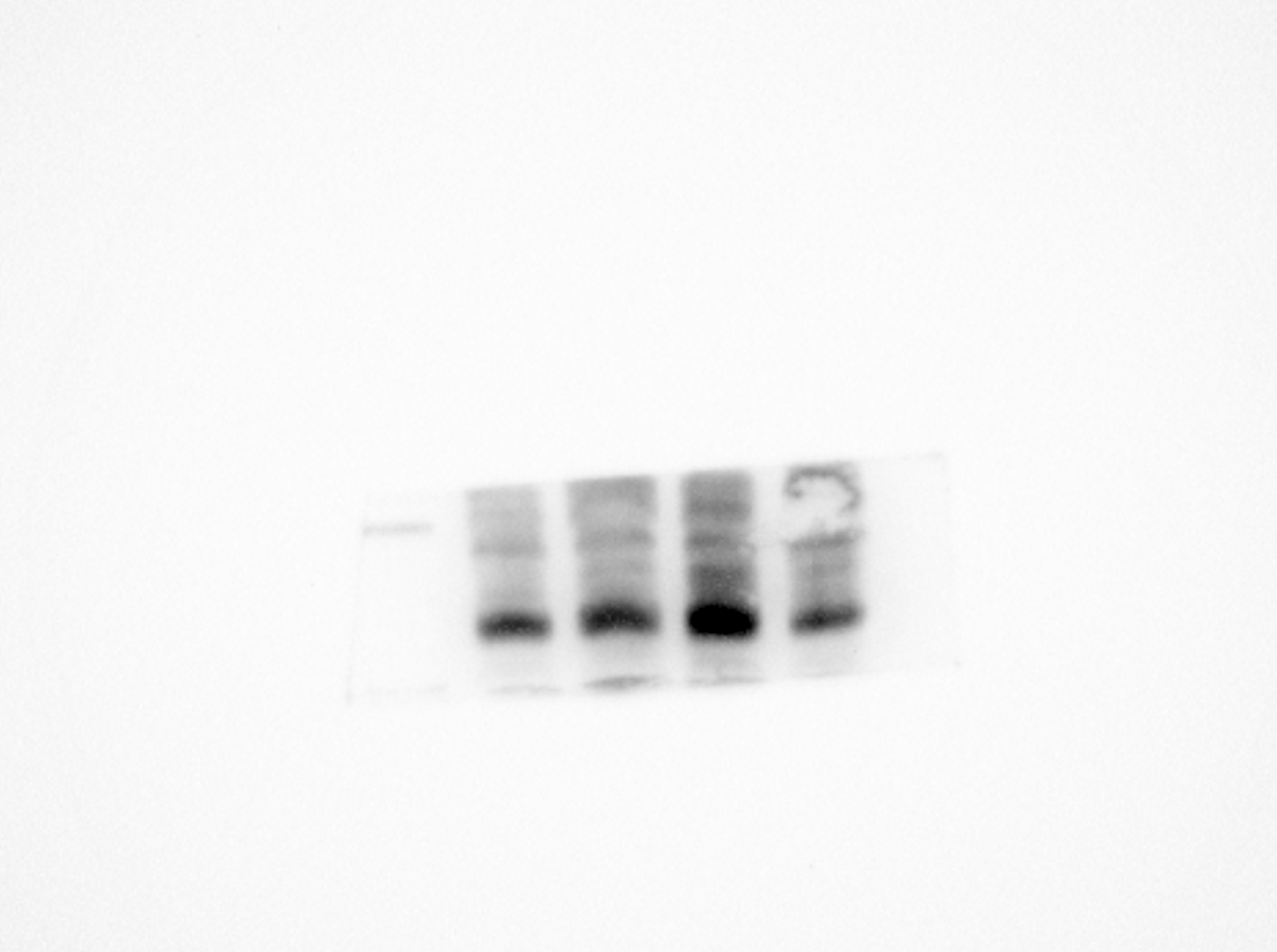

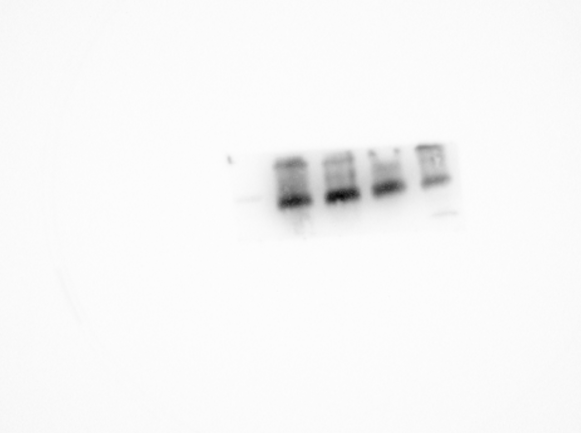

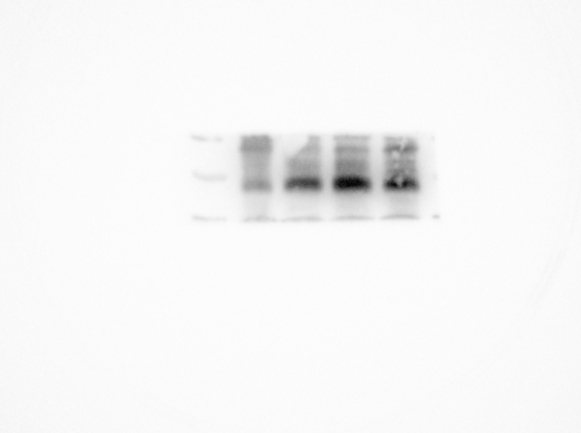

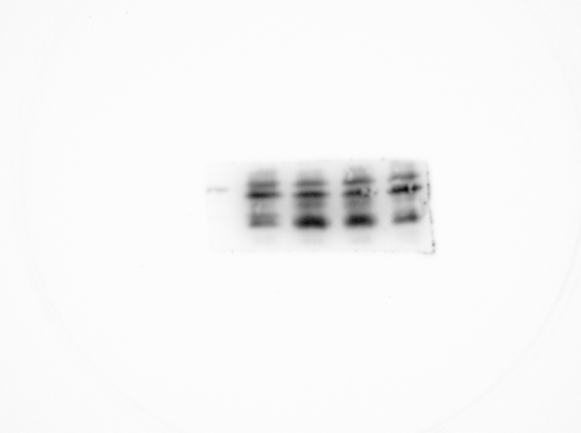


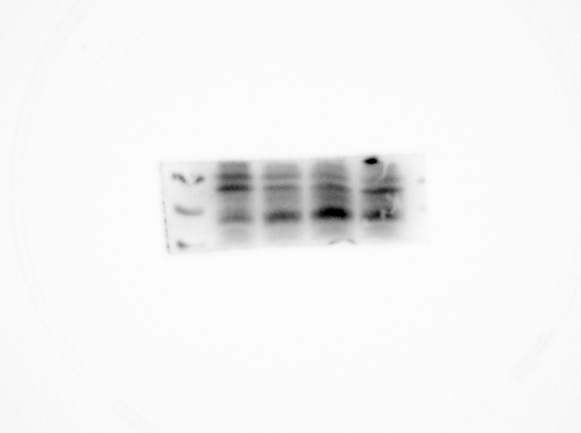

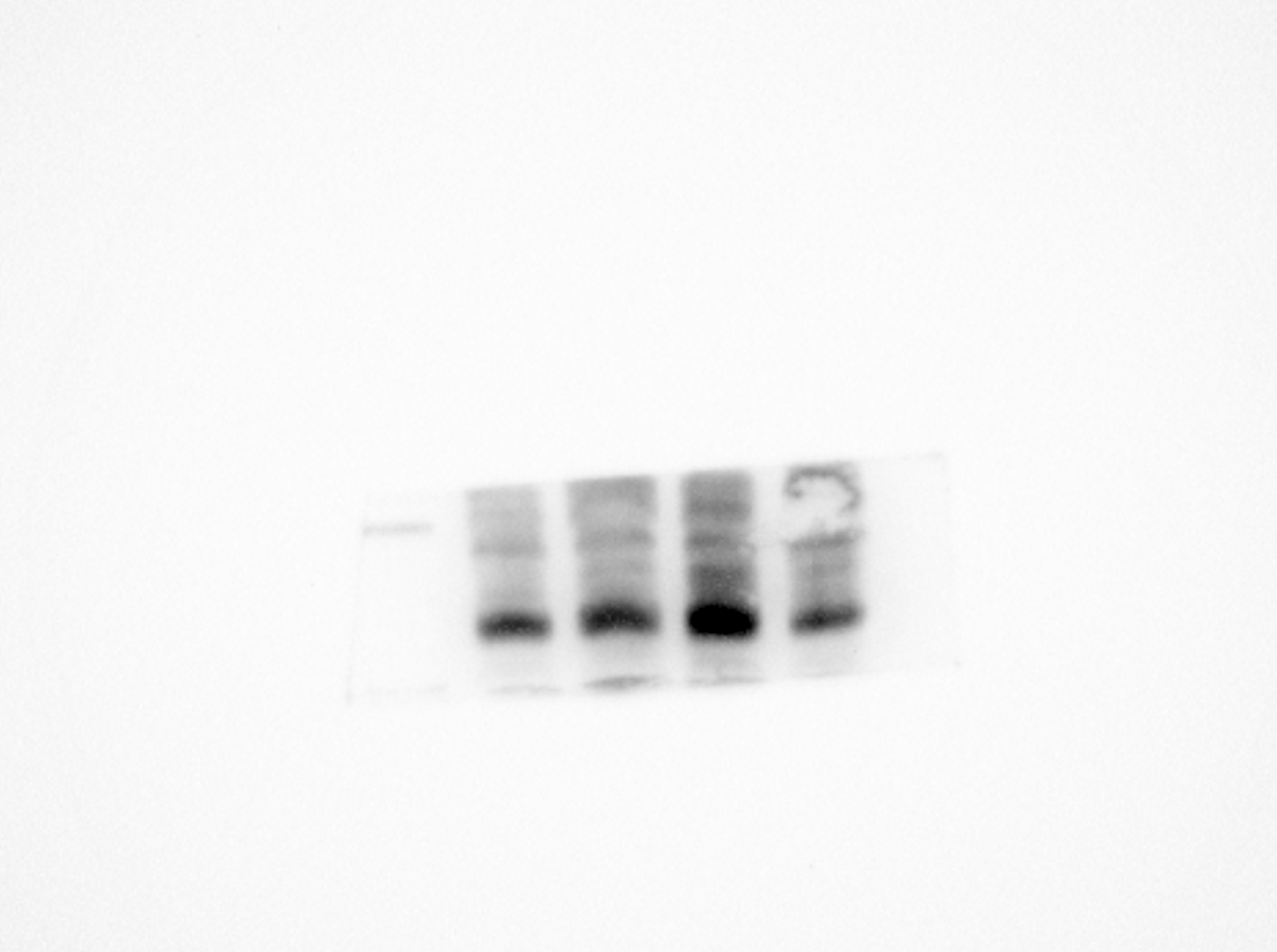

Supplement: Supplementary file 4 — Additional file 4. [file 12915_2025_2472_MOESM4_ESM.docx]
