# Supplementary material for: Rejuvenation of mesenchymal stem cells by human peripheral blood lymphocytes
Source: BMC Biol. 2025 Nov 25;23:370. doi: 10.1186/s12915-025-02472-9 (PMC12750958; doi:10.1186/s12915-025-02472-9)
Supplement: Supplementary file 2 — Additional file 2: Video S1. Real-time monitoring of P10-hAMSC growth for 72 h in culture plate. Video S2. Real-time monitoring of P10-hAMSCs co-cultured with PBLs in culture plate over a 72-h period. [file 12915_2025_2472_MOESM2_ESM.zip › Additional file 2/Additional file 2.docx]

**Supplementary Videos**

**Supplementary video 1. Real-time monitoring of P10-hAMSC growth for 72 h in culture plate.** A total of 272 images were taken at the same position at 100× magnification every 15 min using the fully automatic live cell monitoring system (Etaluma LS850, Etaluma, USA). Later, adobe prime software was used to synthesize the above photos into 30s videos.

**Supplementary video 2. Real-time monitoring of P10-hAMSCs co-cultured with PBLs in culture plate over a 72-hour period.** A total of 272 images were taken at the same position at 100× magnification every 15 min using the fully automatic live cell monitoring system (Etaluma LS850, Etaluma, USA). Later, adobe prime software was used to synthesize the above photos into 30s videos.
